# A spatially structured mathematical model of the gut microbiome reveals factors that increase community stability

**DOI:** 10.1016/j.isci.2023.107499

**Published:** 2023-07-31

**Authors:** Shota Shibasaki, Sara Mitri

**Affiliations:** 1 Department of Fundamental Microbiology, University of Lausanne, Lausanne, Switzerland

**Keywords:** Experimental models in systems biology, Mathematical biosciences, Microbiome

## Abstract

Given the importance of gut microbial communities for human health, we may want to ensure their stability in terms of species composition and function. Here, we built a mathematical model of a simplified gut composed of two connected patches where species and metabolites can flow from an upstream patch, allowing upstream species to affect downstream species’ growth. First, we found that communities in our model are more stable if they assemble through species invasion over time compared to combining a set of species from the start. Second, downstream communities are more stable when species invade the downstream patch less frequently than the upstream patch. Finally, upstream species that have positive effects on downstream species can further increase downstream community stability. Despite it being quite abstract, our model may inform future research on designing more stable microbial communities or increasing the stability of existing ones.

## Highlights

• We built a mathematical model of a gut containing two patches with simulated microbes
• Microbial communities that assemble gradually are more stable than when seeded at once
• Spatial structure promotes stability, as the downstream patch is shielded from invaders
• A downstream community is more stable if species living upstream have positive effects on it

## Graphical abstract

**Figure:**
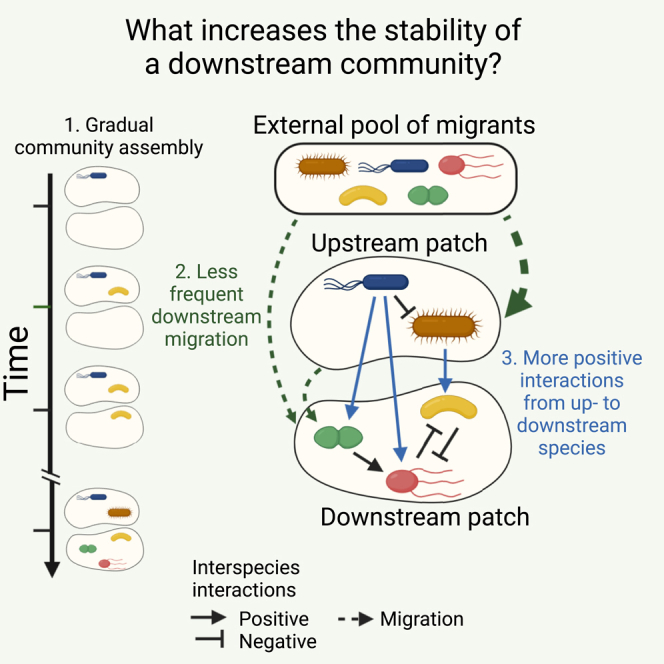

## Introduction

The ecological dynamics of communities of macro- as well as microorganisms depend on whether species are well mixed and equally likely to interact with one another or live in spatially structured environments where species interactions depend on a neighbor’s identity.^1^ Spatial structure can particularly affect the ability of species to coexist, of new species to invade, and thereby the overall diversity of a given community.^2^^,^^3^^,^^4^^,^^5^

Much of the theoretical work on spatial structure in meta-communities considers space as a way to organize species' migration patterns between different patches, and assumes that species exclusively interact locally within each patch. However, it is not hard to imagine that species could affect the growth of others residing in connected patches, thereby affecting the stability of these neighboring communities. In microbial communities, species interactions are often mediated by the environment; for example, where one species consumes resources or produces toxins that affect other species’ growth and death.^6^ If multiple patches are colonized by different species and these environmental factors can flow into connected patches,^7^^,^^8^^,^^9^ a species in a focal patch can affect the growth of another in a connected patch. In some natural environments, such as the gastrointestinal tract^10^^,^^11^ or river ecosystems,^12^^,^^13^ environmental factors flow in one direction, whereby upstream microbial species can affect the growth of others that reside downstream. Environments that are made of discrete patches organized in such a uni-directional chain network are known as hierarchical spatial structures. Here, we are interested in whether and how microbial species residing in upstream “patches” of the gastrointestinal tract can affect the stability of downstream communities.

The relationship between the gut microbiome and human health is now well established,^14^^,^^15^^,^^16^^,^^17^^,^^18^^,^^19^^,^^20^^,^^21^^,^^22^ in particular the role of microbiome stability over time.^23^^,^^24^^,^^25^^,^^26^^,^^27^ This increased understanding has inspired recent ambitions to design gut communities that can improve human health, for example by producing more butyrate or secondary bile acids.^28^^,^^29^^,^^30^ But how to increase the stability of gut microbiomes – whether they have assembled naturally or have been designed for a particular function – is less clear. In particular, the effects of the spatial structure of the gastrointestinal tract on gut microbiome stability has rarely been addressed.

In this manuscript, we construct a mathematical model of a chain network based on the generalized Lotka-Volterra framework composed of two patches, meant to represent a highly simplified gut microbiome. We use it to explore how the stability of a microbial community that resides in the downstream patch is affected by a community living upstream of it. We do not consider the stability of the upstream community because spatial structure does not affect it. Focusing on the downstream community, then, we investigate the factors that affect its stability in two scenarios: first, where the meta-communities assemble naturally through the colonization of sterile patches, and second, where species are randomly allocated to the two patches from the start. We consider the second scenario because a beneficial meta-community may be unlikely to be naturally assembled, but we would still like to inoculate it into the gut, and because statistical analyses are easier than in the first scenario. Our ultimate goal is to manipulate upstream communities in ways that would stabilize downstream communities that are beneficial to the host.

In ecology, many types of stability have been defined,^31^ each with different criteria. Here, we define stability as a lack of change in species composition rather than species abundances over a fixed time frame. This is because we assume that the functions of a community would not collapse as long as the species composition is maintained, although in principle, changes in species abundances could affect community function. We consider a fixed time frame, as it is more representative of a natural scenario, and because we run numerical simulations rather than solving equations to equilibrium. We consider two criteria for stability. One is resistance to invasion^32^^,^^33^: whether an invader species fails to establish and eliminate any of the resident species. This criterion is important because invaders may decrease community function or drive one or more species in the community extinct. Previous studies suggest that species richness (i.e., the number of species in a community) increases the resistance to invasion both in theory^34^ and experiments,^35^^,^^36^^,^^37^ presumably because a species-rich community exploits available resources, making them inaccessible for invaders.^38^ The second stability criterion we consider is resistance to environmental change caused by changes to the upstream community composition. As the upstream and downstream patches are connected, species invasion upstream may change conditions in the downstream patch, which may lead to the extinction of one or more species in the downstream community. We call this stability criterion “structural persistence”. The idea of structural persistence is similar to structural stability, which, in the context of ecology, refers to the volume of the parameter space, typically that of species’ growth rates or the species interaction matrix, where species composition does not change.^39^^,^^40^^,^^41^^,^^42^^,^^43^^,^^44^^,^^45^ Both structural stability and structural persistence analyze the persistence in species composition against perturbations that affect species’ parameters. Such perturbations can be, for example, fluctuations of environmental conditions that a model does not explicitly include.

Our model shows that the presence of an upstream community can stabilize one that is downstream of it. This is mainly because invasion to the upstream patch is unlikely to change the downstream species composition whether an invader establishes or not. The less frequently invaders arrive downstream, the more stable the downstream communities become. Assembled communities were also found to be more stable than designed ones. In addition, we show that increasing the strength of positive interactions from the species residing in the upstream patch to those in the downstream patch increases the stability of the downstream community. These findings may inform on how to increase stability either in naturally assembled communities or those designed from scratch for a target function.

## Results

### Without spatial structure, assembled communities are more stable than designed ones

We are interested in spatially structured communities, where a community in one patch can affect the stability of a second community located in a downstream patch. Before introducing this spatial structure, however, we first define stability and illustrate two methods of sampling communities in a single patch without any spatial structure.

**Figure 1:**
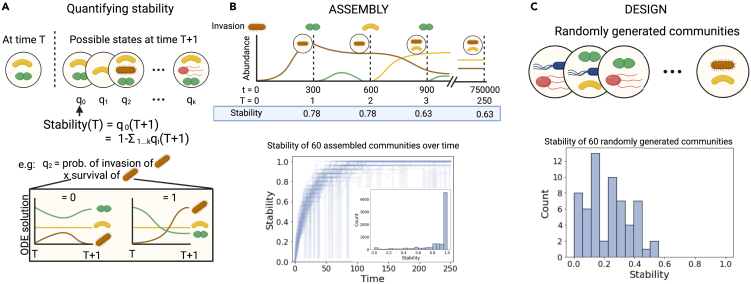
Stability with no spatial structure (A) We define the stability of a community at time step T as the probability $q_{0}\left(T+1\right)$ that the identical species composition is observed at time step T+1 (i.e., after invasion of one species and the stabilization of the dynamics). Species composition can change if an invader establishes and/or one or more resident species go extinct. For example, the probability that a new species establishes $\left(q_{2}\left(T+1\right)\right)$ is decomposed into the probability that a given species invades the community (uniform for all species in the migration pool) and establishes (0 or 1, given by solving the deterministic Equation 1b). See Section Method details for more details. (B) We simulated 60 communities that assembled as in nature through 250 sequential invasion events, where stability is measured at each time cycle T. Between time cycles T, community dynamics play out over 300 timesteps t. The plot shows the changes in stability in the 60 communities over time. The inset shows the histogram of stability over time. We removed data where invasion does not change species composition so that stable communities are not over-sampled. Most of the sampled stability was close to one. (C) In the design scenario, we generated locally stable communities with randomly chosen species composition. Measuring stability in these communities is comparable to measuring at one time point T in the assembly scenario. The plot shows the distribution of the stability of 60 such randomly generated communities. Created with BioRender.com.

We define stability in this manuscript as the probability that species composition (i.e., presence or absence of each species) remains identical after a fixed time period following the invasion of a new species into the system (see Equation 2; Figure 1A). Specifically, the stability of a given community at time cycle T is the probability $q_{0}\left(T+1\right)$ that at time T+1 (equivalent to 300 small time steps t, which Figure S1 shows is long enough for the dynamics to converge), the community has the identical species composition. This is determined by solving the set of ordinary differential equations in Equation 1b describing a generalized Lotka-Volterra model with interaction parameters generated randomly for each community until t=300 after the invasion. In the absence of spatial structure, stability represents the probability that an invader neither establishes nor removes any of the resident species.

We evaluated the stability of simulated communities using two scenarios. The first is the “assembly” scenario, which represents how a natural community would assemble in an initially sterile environment: a new invader species is introduced into the patch at every T time step and the community dynamics are simulated. This process is repeated for a total of T=250 time steps (Figure 1B, top). Without spatial structure, community stability tends to increase over time, and if it reaches one, species composition no longer changes. In some cases, stability suddenly decreases but recovers over the following time steps because the population dynamics may equilibrate very slowly following an invasion and 300 t time steps are not long enough to remove species that would go extinct at equilibrium. While we calculated the stability of many communities in this assembly scenario (each time step in 60 different simulations), these measurements are not all independent as species composition at T depends on that at T−1, which makes it difficult to perform statistical analyses.

To overcome this problem and because it is also practically interesting, we also evaluated communities using a “design” scenario (Figure 1C), in which we randomly generated 60 communities where species coexistence is locally stable (a given species combination could coexist over 300 t time steps, Equation 1b). This scenario represents a case where we may wish to establish a very specifically designed community into a host. In Data S2, we show that species richness increases stability in the absence of spatial structure (Figure S4; Table S1), which is consistent with previous work.^34^^,^^35^^,^^36^^,^^37^ We also find that these designed communities are much less stable than the ones generated by the assembly scenario (median stability is 0.24 and 0.96, respectively, one-sided Wilcoxon rank-sum test: U=−12.099 and $p<10^{-3}$; Cliff’s delta is −0.91), which is also consistent with recent findings.^23^^,^^26^ Higher stability in the assembly scenario is due to higher species richness than in the design scenario (Figures S5–S7).

### Hierarchical spatial structure can increase downstream community stability

**Figure 2:**
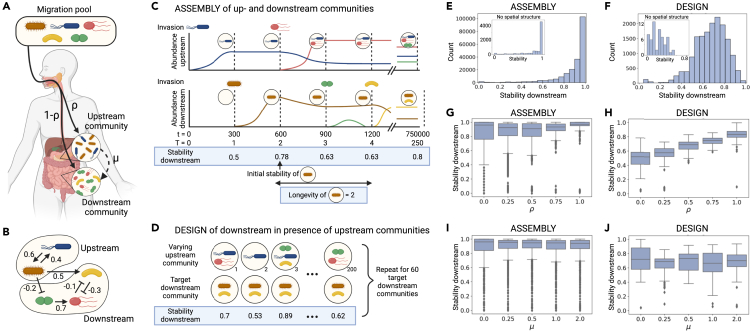
Adding hierarchical spatial structure under two scenarios (A) In our hierarchical spatial structure, we consider the meta-community dynamics with two patches: upstream and downstream. Species can either migrate from the migration pool to (i) the upstream patch, (ii) or the downstream patch with likelihood ρ or 1−ρ, respectively, or (iii) from the upstream patch to the downstream patch with likelihood scaled by μ. (B) An example of upstream and downstream communities and their features (see also Table 1). Here, species richness is two upstream and three downstream, respectively. The upstream community has only positive interspecific interactions and thus their total and mean strength are 1.0 and 0.5, respectively. The downstream community has one positive interspecific interaction and two negative ones. The total and mean strength of positive interactions there are 0.7, while the total and mean strength of negative interactions are 0.4 and 0.2, respectively. In addition, there are a positive and a negative interaction between the two communities: total (and mean) strength of positive interactions from the upstream to the downstream community is 0.5 and that of negative interactions is 0.2. The mean (in-)degrees within the upstream community and the downstream one are 1.0 (upstream: (1+1)/2=1, downstream: (1+2+0)/3=1). The mean degree from the upstream to the downstream is 0.667(=(1+1+0)/3). (C) The assembly scenario is as in Figure 1, but with an upstream community that can affect the downstream community (because of interactions as show in panel B). At each time step, one species migrates into either the up- or downstream community according to parameters ρ and μ. Stability is calculated for the downstream community only, and can change if the composition of the upstream community changes. Community longevity is calculated for a given community composition by counting the number of time steps T in which it persisted: see also Figure S10. We assembled 60 such communities for each parameter set (ρ,μ). (D) In the design scenario, 60 target downstream communities were generated for each parameter set (ρ,μ), and for each target, we generated 200 upstream communities and analyzed the stability of the downstream community using logistic regression. (E–J) The stability of the downstream communities sampled from the assembly (E, G, and I) and design (F, H, and J) scenarios. The top panels (E and F) represent the stability distribution under hierarchical spatial structure (with insets showing the distribution with no spatial structure), while the middle (G and H) and bottom panels (I and J) show the distributions given migration parameters ρ (migration from the migration pool to the upstream patch versus to the downstream) and μ (migration from the upstream patch to the downstream patch), respectively. Created with BioRender.com.

Next, we consider a chain network composed of two patches, which can be seen to represent a simplified gut environment and focus on the stability of the downstream community. The stability of the upstream community corresponds to the case without spatial structure, because it is not affected by downstream species (see Data S2). Having introduced spatial structure, invaders can colonize the two patches in three different ways: (i) from outside of the system (the migration pool) to the upstream patch, proportional to ρ, (ii) from the migration pool to the downstream patch, according to 1−ρ, and (iii) from the up- to the downstream patch, depending on μ (Figure 2A). The two parameters ρ and μ affect the probability of invasion and thereby the stability measure (Figure 1A). If μ is larger (smaller) than 1, migration from up- to downstream is more (less) likely to occur than migration from the migration pool (see section Method details for more details). As it is unclear what realistic values of ρ and μ would be, we analyze their effect on stability across the whole parameter range.

Downstream community stability is decomposed into its ability (1) to resist the arrival of invaders downstream (resistance to invasion) and (2) to persist despite environmental changes that occur due to the arrival of migrants upstream (structural persistence) (Equation 2). An upstream community can affect both types of effects: If we assume that the overall invasion rate is the same regardless of spatial structure (one invasion event at every time step T) and if ρ>0, the upstream patch decreases the rate of direct invasions into the downstream patch. Second, if species invade the upstream patch, they can affect the downstream species. This is implemented by adding interaction terms to the gLV equations describing the dynamics of the downstream species that depend on upstream species abundances (Equation 1b). This is meant to represent situations where upstream species affect the downstream community, for example by producing resources that flow into the downstream patch (Figure 2B). In the spatially structured model then, downstream stability can drop even after it has reached one because species that successfully invade the upstream patch can change the growth rates in the downstream patch and reduce downstream stability. This would never occur in the absence of the upstream patch.

To quantify the effect of spatial structure on stability, we again consider our two scenarios. In the assembly scenario, we sampled 60 time-series of meta-communities until T=250 for a given parameter set (ρ,μ) but removed communities where invasion does not change species abundances to avoid oversampling stable communities. This resulted in n=209260 downstream community stability measurements whose distribution is skewed close to one (median: 0.949, Figure 2E), with stability increasing with time (Spearman’s correlation coefficient: 0.671 and $p<10^{-3}$, Figure S2). As this resembles the patterns with no spatial structure (Figure 1B, inset of Figure 2E), the assembly scenario likely generates stable communities regardless of the spatial structure. Figure S10 also shows that the downstream communities are maintained for longer when the initial stability (i.e., the downstream stability when the focal community is generated) is larger. This suggests a benefit to designing initially stable downstream communities.

In the design scenario, we randomly sampled 60 downstream communities and exposed them to 200 randomly generated upstream communities for a given parameter set (ρ,μ) (Figure 2D): in total, we generated 60×200×25=300,000 meta-communities. We then removed the data where one or more species in the generated meta-communities went extinct, resulting in a total of n=16,160 stability measurements. Indeed, it is difficult to generate locally stable meta-communities, as many randomly generated meta-communities lose species in either or both patches before the dynamics stabilize (Figure S3, Data S3 discusses coexistence in the meta-community). As in the absence of spatial structure, downstream communities in the assembly scenario are more stable than in the design scenario (one-sided Wilcoxon rank-sum test: U=161.888 and $p<10^{-3}$; Cliff’s delta is 0.76, Figures 2E and 2F). However, the presence of an upstream community increased the stability of a downstream one in the design scenario (median of 0.692 and 0.24 with and without spatial structure, respectively; maximum stability without spatial structure <0.6, Figures 1C and 2F including inset). This is in part because the probability of migration into the downstream patch is reduced if ρ>0, resulting in less frequent perturbations (Figure 1A). The upstream community thereby “absorbs” invaders by reducing the rate at which species arrive downstream. This is due to the way we choose to model migration (using ρ and 1−ρ). But even when ρ=0 (i.e., no species invade upstream and thus structural persistence does not affect stability and invasion frequency is as with no spatial structure), stability was significantly higher than with no spatial structure (medians of stability are 0.4 and 0.24, respectively; one-sided Wilcoxon rank-sum test: U=8.086 and $p<10^{-3}$; Cliff’s delta is 0.61). This result indicates that upstream communities can affect the downstream resistance to invasion by other ways than absorbing invaders. We investigate this in section Causal inference analysis finds features that affect stability. In contrast, when (ρ,μ)=(1,0), all invasions occur upstream and stability depends only on structural persistence. In this case, the median stability is 0.96, and the downstream community is more stable than without spatial structure (median stability: 0.96 and 0.24, respectively; one-sided Wilcoxon rank-sum test: U=10.599 and $p<10^{-3}$; Cliff’s delta is 0.82), suggesting that invasion to upstream communities is unlikely to change downstream species composition.

### Migration parameters affect stability of downstream communities

Host physiology (e.g., stomach acidity) may influence the likelihood of microbes colonizing an upstream versus a downstream patch, or the likelihood of microbes migrating from one patch to the other. These likelihoods are represented in our model by the two migration parameters, ρ and μ, and we now investigate how they affect downstream stability (Figures 2G–2J).

In both sampling scenarios, ρ increased downstream stability while μ decreased it (logistic regression coefficients for ρ and μ in assembly: 0.439 and −0.0426 respectively, and for ρ and μ in design: 0.557 and −0.0585, all p values $<10^{-3}$). It appears, then, that migration into the downstream community reduces stability more than changes to the upstream community do. The greater magnitude of the coefficients of ρ compared to μ, however, show that migration from the outside species pool has stronger effects on stability than migration from up- to downstream. Indeed, Tables S9 and S10 (see also Data S6) show that increasing ρ stabilizes the downstream communities more than any other community feature that we analyzed, including μ. This suggests that the invasion of new species downstream is what decreases community stability the most. Intuitively, downstream invaders can change downstream species composition in two ways: (i) by successfully colonizing or (ii) by driving one or more resident species extinct, even if they do not colonize themselves. On the other hand, upstream invaders can only change downstream species composition if they greatly affect upstream abundances and thereby the downstream environment. It appears then that compared to direct downstream colonization, successful upstream colonization is less likely to change downstream species composition.

### Causal inference analysis finds features that affect stability

So far, we have learned that the stability of a downstream community can be increased by placing another community upstream of it to decrease the arrival of migrant species that may destabilize it. This is helpful, but if we wish to design a specific target downstream community that is stable (the design scenario), or stabilize a naturally assembled community in the downstream patch (the assembly scenario), other features of the system that affect downstream stability may also be worth controlling, such as the species composition upstream, or how they interact with the downstream species. We also know that reducing migration is not the only stabilizing force of the upstream community, as we showed above that the upstream community still increases downstream stability when all migrants land in the downstream patch.

To determine which features make downstream communities more stable, we compiled a list of such community features in Table 1 (see also the caption of Figure 2B for examples) and repeatedly sampled the design scenario to generate sufficient data. We first applied logistic regression models to establish which features might be interesting to explore further (see Data S5; see also Figures S2–S8). Interestingly, although species richness increases stability in the absence of spatial structure, it does not even correlate with stability in the spatially structured model (Table S4–S7; see also Figure S8). Instead, all the models revealed three features that correlated with downstream stability: the mean degree (number of other species each interacts with) between the up- and downstream communities, ρ, and μ.

**Table 1:** List of analyzed features

Feature or symbol	Explanation
Total positive upstream	Total strength of positive species interactions upstream
Mean positive upstream	Mean strength of positive species interactions upstream
Total negative upstream	Total strength of negative species interactions upstream
Mean negative upstream	Mean strength of negative species interactions upstream
Mean degree upstream	Mean degree upstream
Richness upstream	Species richness upstream
Total positive downstream	Total strength of positive species interactions downstream
Mean positive downstream	Mean strength of positive species interactions downstream
Total negative downstream	Total strength of negative species interactions downstream
Mean negative downstream	Mean strength of negative species interactions downstream
Mean degree downstream	Mean degree downstream
Richness downstream	Species richness downstream
Total positive *trans*	Total strength of positive species interactions from up- to downstream
Mean positive *trans*	Mean strength of positive species interactions from up- to downstream
Total negative *trans*	Total strength of negative species interactions from up- to downstream
Mean negative *trans*	Mean strength of negative species interactions from up- to downstream
Mean degree *trans*	Mean in-degree from up- to downstream
ρ	Migration from the migration pool to upstream (1−ρ: to downstream)
μ	Migration from up- to downstream

Note that some features are only used for predicting stability, see Data S5.

**Figure 3:**
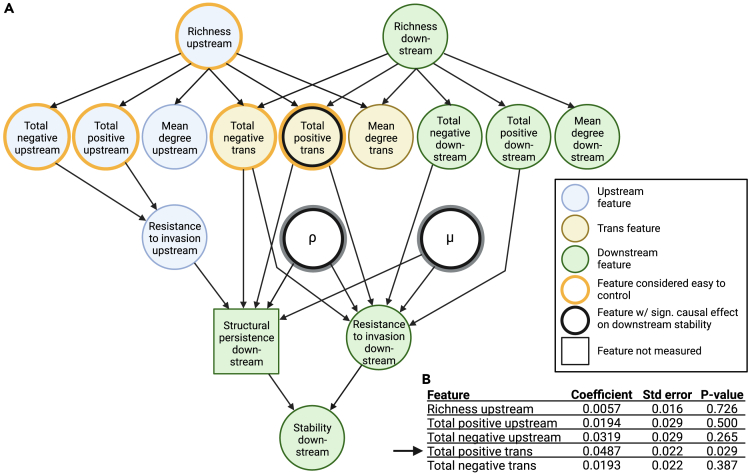
A full model of the causal diagram and resulting estimates (A) The assumed causal diagram is represented by a directed acyclic graph. Circles filled in blue, yellow and green are features related to the upstream community, the transition between the two communities and the downstream community, respectively. The features with a thick orange border are the five features we expect to be able to control externally. Features with a thick black border were found to have significant causal effects on downstream stability. The unobserved factor in this study, resistance to invasion in the upstream community, is written in a box. The main text outlines the rationale behind this model, and features are explained in Table 1. (B) Summary statistics of estimated causation on stability via logistic regression analysis. Only “total positive *trans*” had a significant causal effect on stability and is therefore highlighted with a black arrow and with a thick black border in panel (A). The effects of ρ and μ are shown in Figure 2 and statistics in the causal inference model in Table S9.

Correlation does not imply causation, however, and we know that many features do not only correlate with downstream stability but also with one another (Figure S13), which may obscure their relationships. To infer causality, we applied causal inference^46^^,^^47^^,^^48^ whereby different causal diagrams can be analyzed to clarify which community features a logistic regression model should include to infer causal effects. For example, in Data S6, we use several causal diagrams to analyze whether the mean degree between up- and downstream communities has a causal effect on stability and found no significant effect, even if this feature correlates significantly with stability (Figure S13). On the other hand, the causal effects of ρ and μ were consistent in all models (Data S6). These initial analyses, together with intuition and features expected to affect community stability based on previous work resulted in the diagram in Figure 3A (see section Qualification and statistical analysis).

In the rest of the main text, we assumed the causal diagram shown in Figure 3A and focused on five features (other than ρ and μ) that we consider to be reasonably easy to manipulate in practice since they are properties of the upstream community, which is more “accessible”: species richness upstream, total strength of positive and negative interactions within the upstream community, and the total strength of positive or negative interactions from the up- to the downstream community. The causal effects of the five features on stability in the data from the design scenario are summarized in Figure 3B. The total strength of positive interactions from up- to downstream species was the only one of the five features that significantly increased stability, with the largest coefficient (see Data S6 for details).

### Positive interactions across patches increase downstream community stability

The causal inference analysis allows us to quickly pinpoint interesting features, but as it is based on several assumptions, we next verified the causal effect of total positive interactions from the up- to the downstream species with three additional analyses. First, we explored whether this feature was also important in the assembly scenario, where we found downstream communities to be very stable (Figures 2E and 2F). Indeed, the total strength of positive interactions from up- to downstream species was significantly greater in the assembly (median: 5.944) than in the design scenario (median: 1.132, one-side Wilcoxon rank-sum test: U=95.616, $p<10^{-3}$; Cliff’s delta is 0.42), where downstream communities were less stable.

**Figure 4:**
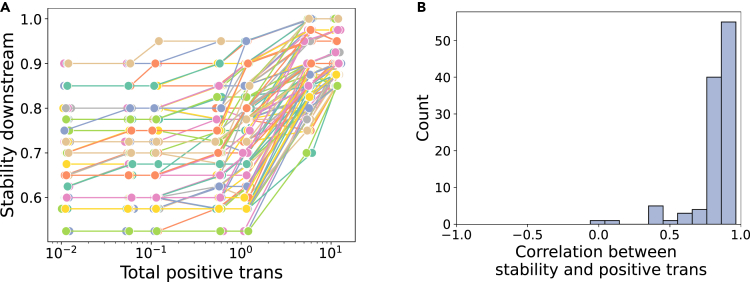
Manipulating positive interactions from upstream to downstream We manipulated only the total strength of positive interactions from the upstream to the downstream communities while keeping other community features constant. (A) Each line represents a different meta-community (n=110). (B) We calculated Spearman's correlation coefficient between stability and the total strength of positive interactions from the upstream to the downstream community in each meta-community. Except for one meta-community, downstream stability positively correlates with the total strength of positive interactions and the correlation coefficients are large (>0.8) in many cases.

Second, we generated 110 meta-communities where species compositions in the up- and downstream communities do not overlap, which allowed us to change the total strength of positive interactions from up- to downstream without changing other community features. Figure 4 shows that the downstream communities become more stable as the total strength of positive interactions from up- to downstream is increased. In this analysis, however, interactions from down- to upstream species are assumed to be negative to keep the assumption that all interspecific interactions follow a normal distribution with mean zero. The increase in stability may then be because colonizing species experience negative interactions on arrival in the downstream patch, rather than the positive interactions we are interested in. To remove this bias, we performed a similar analysis while assuming species interaction effects from down- to upstream species are zero and found that the strength of positive interactions from up- to downstream species tends to correlate positively with stability, although the effect is much weaker than in our first analysis (see Data S7, compare Figures 4 and S14).

**Figure 5:**
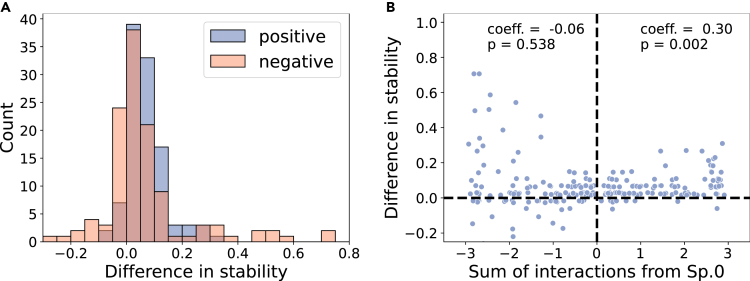
Changes in stability in the pseudo-structured model In this analysis, we removed the spatial structure but assumed species 0 whose growth is not affected by the remaining species. We manipulated species 0’s interactions with three resident species in 240 simulation runs, and measured the stability of the resident communities with and without species 0. We removed some communities where the presence/absence of species 0 affects the coexistence of the resident species. (A) Histograms of difference in stability (stability of the residents with species 0 - without species 0). When species 0 has either positive or negative effects on the three resident species, stability increases (one-sided Wilcoxon signed-rank test: positive interactions: N=108, T=5687.0, and $p<10^{-3}$, negative interactions: N=119, T=5646.0, and $p<10^{-3}$). This may be because species richness increases stability without spatial structure (Table S1). (B) Testing for correlations between the total strength of interactions from species 0 to the three resident species and the difference in stability in its presence/absence (as in panel A). We find no significant correlation with negative interactions (Spearman correlation test, N=119, the correlation coefficient is ρ=−0.06, p=0.538), but the sum of positive interactions from species 0 correlates positively with the increase in stability in its presence (Spearman correlation test, N=108, ρ=0.3, p=0.002).

Finally, we ran 240 additional simulations without spatial structure but we assumed specific species interaction matrices: one species (species 0) is not affected by the rest as if it were upstream while the other species resided downstream. In this pseudo-spatially structured model, we assumed three-species resident communities (without species 0), and we manipulated how species 0 interacts with the three resident species while the remaining interactions (except for those affecting species 0) follow a random matrix. The interactions from species 0 to the three resident species correspond to the interactions from up- to downstream communities in the structured model. We compared the stability of the resident communities with or without species 0, and over the total strength of interactions from species 0 to the resident species. First, we find the presence of species 0 increases stability regardless of the sign of its interactions (one-sided Wilcoxon signed-rank test: positive interactions: W=7061.0, and $p<10^{-3}$; the rank-biserial correlation is 0.95, negative interactions: W=5646.0, and $p<10^{-3}$; the rank-biserial correlation is 0.59, Figure 5A), which may be because species richness increases stability in the absence of spatial structure (Table S1). However, we also find that stability correlates positively with the sum of positive interactions from species 0 (Figure 5B), supporting our previous results. Taken together, we conclude that positive interactions from up- to downstream species tend to stabilize downstream communities.

## Discussion

As it is now well established that the gut microbiome can affect host health, recent efforts have turned to manipulating and designing beneficial gut microbial communities.^28^^,^^29^^,^^30^ Maximizing the beneficial effects of natural or designed communities relies heavily on the ability to stabilize their composition and function, yet we know little about how the spatial organization of the gut affects microbial community stability, particularly in the large intestine where most microbes live. In this manuscript, we built a mathematical model to simulate the ecological dynamics in two connected “patches”, meant to represent a simplified gut with different areas flowing into one another, and analyzed the stability of the community residing in the downstream patch. This has revealed three main factors that affect the stability of a downstream community.

First, we found that the way in which a community assembles strongly affects its stability. We simulated the natural process by which microbial communities assemble by invading one species at a time, and compared it to simulations where we chose a set of species (presumably ones that together have a beneficial function) and placed them in the two patches. Assembled communities were significantly more stable than designed ones (Figures 2E and 2F), which is consistent with previous studies showing temporal stability of gut microbiomes.^23^^,^^26^ Indeed, designing an upstream community that did not collapse the target downstream communities was challenging (Figure S3, see also Data S3).

The second factor that affects stability downstream is which patch species invade. In our simulations, species invaded the system at a constant rate, but could either invade the up- or downstream patch from outside or migrate from the up- to the downstream patch. The most stable downstream communities were observed when outside invasion into the downstream patch was minimized, suggesting that invasion events were more destabilizing for the downstream community compared to colonization events upstream that could indirectly affect downstream species composition. While it is unclear how easy it is to modify species migration in a real gut, they may have evolved to include barriers, such as an acidic stomach to keep invaders from arriving downstream.

The third result from our model is that downstream stability can be increased if species upstream have positive effects on those downstream. Such positive effects can occur if upstream species produce metabolites that downstream species can grow on, or if they absorb toxins that would otherwise inhibit downstream species. Intuitively, increasing such positive effects can prevent the extinction of the downstream species when invasions occur in either patch. In a related study, Qian and Akçay^33^ developed a model that corresponds to our assembly scenario without the spatial structure, and found that mutualistic interactions (bidirectional positive interactions between a pair of species) reduced colonization by invaders and extinction of resident species because they increase resident species abundances. An invader is thereby faced with more competitors that suppress its growth rate, and a smaller likelihood that the residents would go extinct. This logic also explains why positive interactions from upstream to downstream species increase stability in our study: stronger positive interactions increase the population abundances downstream, increasing resistance to invasion.

In practice, one way to increase these positive effects would be to increase species richness upstream. However, increasing upstream species richness may (1) collapse coexistence in the upstream patch according to May,^49^ (2) drive one or more species extinct in the downstream patch, (3) change other community features (Figure S15, Data S8), and (4) species richness alone cannot increase stability (Figure 3B). To stabilize the downstream community, therefore, we need to carefully choose species to introduce into the upstream community, such that (a) they do not drive other species extinct in either of the two patches, and (b) they have positive effects on the downstream species while having negligible effects on other community features (Figures 4 and S14). As discussed in Data S3, deriving conditions to satisfy (a) in general is difficult. Suppose we could increase the positive effects from the upstream to the downstream by increasing the upstream species richness. In that case, we can also expect that the upstream stability (i.e., the resistance to invasion upstream) increases as suggested by previous studies without spatial structure^35^^,^^36^^,^^37^ (see also Data S2).

It is important to note that our measure of stability in a downstream community within two connected patches differs substantially from other measures of stability in the field: our stability is composed of resistance to invasion^32^^,^^33^^,^^34^^,^^35^^,^^36^^,^^37^ and structural persistence (persistence despite environmental changes caused by upstream invasion events).

Local stability, whose relationship to species interactions has been an important research focus,^49^^,^^50^^,^^51^ considers small perturbations in population abundances and is binary (i.e., a community is either stable or not), such that locally stable communities have complete resistance to invasion. The measure of stability in our model is less strict: if only few species can invade a community (i.e., local stability =0), it is still considered to be highly stable (but <1). This difference has led to some apparent inconsistencies between our results and previous studies. First, in contrast to May’s seminal work,^49^ we find that species richness increases stability in the absence of spatial structure (Table S1). This is because invaders experience more negative interactions in total in a richer community, which reduces invasion success (higher stability in our model). But as long as invasion success is >0, a community is locally unstable. Second, positive interactions increase stability in our study (Figure 3, with spatial structure), but decrease it in the model by Coyte et al. ^51^ In our study, positive interactions from up- to downstream increase population sizes downstream, making species less likely to go extinct. The local stability criterion instead requires abundances to remain finite, which is less likely to hold if a community contains reciprocal positive interactions. In a hierarchically structured system like ours, though, positive interactions are uni-directional.

In addition to perturbations caused by invasions, the presence of upstream species means that downstream species’ growth can also be affected, which can be seen as changes in growth parameters due to environmental changes. Neither local stability nor resistance to invasion can capture these perturbations. Our stability measure therefore also includes what we are calling “structural persistence”. Structural persistence is similar to structural stability^39^^,^^40^^,^^41^^,^^42^^,^^43^^,^^44^^,^^45^ because both consider how perturbations change parameter values, typically growth rates. While structural stability assumes sudden and permanent changes in parameter values, structural persistence assumes gradual changes over time because the realized intrinsic growth rates change depending on the upstream dynamics. In addition, changes in parameter values can be biased in structural persistence because of how parameter changes depend on changes in the upstream communities. In contrast, structural stability considers uniform changes in parameter space. Structural persistence would be, therefore, identical to structural stability if (a) upstream dynamics equilibrate very quickly after an invasion, and if (b) changes upstream can affect parameters of downstream species uniformly; this would happen, for example, if all species were equally likely to invade the upstream patch.

To what extent can the results of our model help us to understand gut communities and their stability? Clearly, our model is quite abstract. It considers only two discrete patches, with each patch containing just a few species, and ignores any metabolic function of the microbes or any host effects. Nevertheless, focusing only on the interactions between species in just two patches makes clear predictions on what affects community stability that one could explore further with a scaled-up model, with different ways of modeling interactions and with different measures of stability.

Coupling our model with prior knowledge about different areas of the gastro-intestinal tract may help to interpret its results. For example, we may consider the two patches to be located in the small and large intestine, respectively, in which case species migration from up- to downstream would be quite frequent (large μ), and the frequencies of migration from outside may be similar for both patches (i.e., ρ around 0.5). Our model then predicts greater stability downstream in the large intestine if species located in the small intestine have strong positive effects on those downstream. Instead, the upstream community may be an oral microbiome and the downstream community that in the large intestine, which would translate to more frequent invasion up- than downstream (large ρ) and little migration between patches (small μ). In this case, the assumption that the same species interact similarly in the two patches may be violated, because there would be many intermediate communities that might influence species interactions.

In summary, we investigated the stability of a downstream patch within a simplified gut microbiome model using meta-community dynamics. We propose three ideas to stabilize downstream communities: First, naturally assembled communities are more stable than randomly generated ones; second, if possible, invasion from outside the meta-community should occur more frequently into the upstream rather than the downstream patch; and third, one should design an upstream community such that its species have strong positive effects on the species residing in the downstream patch. Finally, while the model was constructed with the gastro-intestinal tract in mind, its results could be applied to other hierarchically structured communities, such as river ecosystems, or wastewater treatment plants.

### Limitations of the study

One limitation of our study is that the results are based on the gLV model. Although gLV models are widely used in ecology due to their simplicity, Momeni et al.^52^ show that these models fail in capturing several pairwise microbial interactions. This is because many microbial interactions are mediated by chemical compounds such as resources and toxins while gLV models represent phenomenological species interactions. Due to these chemically mediated interactions, the strength and sign of microbial species interactions can change depending on environmental conditions.^6^^,^^53^^,^^54^^,^^55^ If microbial interactions are environmentally mediated, downstream species interactions may change depending on upstream species composition. In such cases, the gLV is not valid. One way to overcome this problem is by building a consumer-resource (CR) model, where the dynamics of species, resources, and toxins are explicitly included. However, we chose the gLV model because a CR model is more complex. The gLV model requires only two types of parameters: the intrinsic growth rate and the species interaction matrices, and one can easily obtain species interactions from the interaction matrices. A CR model, on the other hand, requires parameters for consumption or production rates of resources and toxins, biomass yields, and the numbers of resources and toxins in the system. Because the dynamics of resources and toxins are rarely available in empirical data, estimating (probability distributions of) these parameter values is difficult. In addition, species interactions in a CR model depend on the resource and toxin concentrations at a given time point. This makes it difficult to infer how species interactions affect stability. Recently, Dedrick et al.^56^ suggested that a gLV model can be a good approximation when species' growth is limited by multiple types of resources. Based on Venturelli et al.*,*^57^ a gLV model can be a good candidate model to represent dynamics in the human gut microbiota.

## STAR★Methods

### Key resources table

**Table:** 

REAGENT or RESOURCE	SOURCE	IDENTIFIER
**Others**		

Codes	Zenodo	https://doi.org/10.5281/zenodo.7959283
Simulation data	Zenodo	https://doi.org/10.5281/zenodo.7959283

### Resource availability

#### Lead contact

Further information should be directed to and will be fulfilled by the lead contact, Sara Mitri (sara.mitri@unil.ch).

#### Materials availability

This study did not generate any new materials.

### Method details

#### Dynamics within each patch

In this manuscript, we consider meta-community dynamics in the simplest chain network composed of two patches: an upstream and a downstream patch. While species interact within each patch, species in the upstream patch can also affect the dynamics in the downstream community because chemical compounds flow from the upstream patch into the downstream patch. We do not model these substrates explicitly, but represent them by interaction terms $a_{ik}$. For example, if an upstream species k consumes a resource that a downstream species i needs, the upstream species will have a negative effect on the growth of that species in the downstream patch (negative $a_{ik}$). In contrast, if species k in the upstream community produces beneficial byproducts, it has positive effects on the species i’s growth in the downstream patch (positive $a_{ik}$). Without migration of species, we can represent the dynamics of species i’s abundance (i=1,...,N) in patch j (where j=1,2 represents up- and downstream patches, respectively) using a generalized Lotka-Volterra (gLV) model:

(Equation 1a) $$\frac{dx_{i1}}{dt}=x_{i1}\left(r_{i1}+\sum_{k=1}^{N}a_{ik}x_{k1}\right)$$

(Equation 1b) $$\frac{dx_{i2}}{dt}=x_{i2}\left\{r_{i2}+\sum_{k=1}^{N}a_{ik}\left(x_{k1}+x_{k2}\right)\right\}\text{,}$$

where N is the maximum number of species in a patch, $x_{ij}$ represents species i’s abundance in patch j, $r_{ij}$ is the intrinsic growth rate of species i in patch j, and $a_{ik}$ represents the net interaction from species k to i. Here, we assume that species’ intrinsic growth rates can differ between the two patches due to environmental conditions that species cannot change (e.g., temperature), while species interactions remain constant between patches. Although gLV models represent species interactions phenomenologically, we assume that all species interact with one another in indirect ways: secreting or absorbing chemical compounds which are not explicitly written in this model. We assume that the environmental difference between up- and downstream is enough small that species interactions are consistent between the two patches. For simplicity, we scaled the interaction parameters so that $a_{ii}=-1$ for all i so that the abundance of species i in patch j is $x_{ij}^{*}=r_{ij}$ at equilibrium in mono-culture. See Data S1 for more details and how we could fit this model to empirical data. For simulating cases without spatial structure, we used Equation 1b with $x_{k1}=0$ for all k, ρ=0, and μ=0.

#### Strength of interactions and degree

In the main text, we used the total or mean strength of positive or negative interactions as community features. Within the upstream or downstream patch, the total strength of positive (or negative) interactions is the sum of absolute values of the realized positive (or negative) interactions: i.e., we consider the sum of $\left|a_{ik}\right|$ where $a_{ik}>0$ (or $a_{ik}<0$ for the negative interactions), $x_{ij}>0$, $x_{kj}>0$, and j=1 (upstream) or 2 (downstream). The total strength of positive (or negative) interactions is calculated in a similar way, but we consider realized positive (or negative) interactions from upstream species to downstream species (i.e., the sum of $\left|a_{ik}\right|$ when $x_{i1}>0$ and $x_{k2}>0$). When we measure the mean strength of positive (or negative) interactions within or between patches, we divide the total strength of positive (or negative) interactions by the number of realized positive (negative) interactions there. See Figure 2B as examples. We did not take into account the population sizes in the analysis of the strength of interactions, although species i affect species j’s growth in the form of $a_{ji}x_{i}$. This is because the scale of the population abundance x can vary in each simulation depending on the values of the sampled growth rates and interaction terms.

We also measured mean degree as another community feature. We omitted the intra-specific interactions in the calculation of degree because all species have such interactions. Because species interaction networks are directed, one can consider in-degree (i.e., how many species affect the focal species’ growth) and out-degree (i.e., how many species’ growth the focal species affects). In this study, we choose in-degree because we are interested in the effects species receive from other species. The mean degree upstream (downstream) is obtained by the sum of in-degree within the patch (i.e., the number of interactions from present species in the same patch to the focal present species) divided by the number of present species there. The mean degree trans is, on the other hand, calculated by the sum of the in-degree between patches (i.e., the number of interactions from present upstream species to a focal downstream species) divided by the number of present species downstream.

#### Defining stability

This manuscript investigates the effect of hierarchical spatial structure on the stability of downstream communities. By assuming rare but regular species migration, our model has two time scales: the short time scale t, when we consider the growth of species in the ordinary differential equations Equations 1a and 1b, and the long time scale T when we consider species invasion and stability of the communities. We ran the simulations given by Equations 1a and 1b for a long time T using solve_ivp function with LSODA in scipy packages,^58^ after which a new species invades into either upstream or downstream communities (but does not necessarily establish).

We define stability at T as the probability that downstream species composition (i.e., presence/absence of each species) does not change after one species invades either the up- or downstream patch (Figure 1A). Without loss of generality, we can denote $q_{0}\left(T+1\right)$ as the probability that the downstream species composition is identical to that at T. Then,

(Equation 2) $$\begin{array}{cc} \text{Stability}\left(T\right) & \equiv q_{0}\left(T+1\right)\\ & =1-\sum_{i\neq 0}q_{i}\left(T+1\right)\text{,} \end{array}$$

where $q_{i}\left(T+1\right),i\neq 0$ represents the probability that the downstream species composition changes to state i at time step T+1. State i=0 is the same state as in time step T.

Our measure of stability for the downstream community can be divided into two criteria: resistance to invasion and structural persistence (persistence following environmental change). Resistance to invasion is the probability that an invader species fails to colonize the downstream community and to exclude any resident species (i.e., the downstream species composition is maintained). We use Ri(i,T) to represent whether downstream species composition at time step T is maintained or not when species i invades the downstream community:

(Equation 3) $$\text{Ri}{\left(i,T\right)}_{=}\left\{ \begin{array}{cc} 1 & \left(\text{maintained}\right)\\ 0 & \left(\text{otherwise}\right)\text{.} \end{array}\right.$$

We do not care about whether the upstream species composition changes or not here.

Structural persistence represents the maintenance of the downstream community composition when a species invades the upstream community, changing the environment, i.e. the realized growth rates of downstream species. Such upstream changes can thereby cause the extinction of one or more downstream species. This effect can be clarified by rewriting Equation 1b as follows:

(Equation 4) $$\frac{dx_{i2}}{dt}=x_{i2}\left\{{\hat{r}}_{i2}\left({\vec{x}}_{1}\right)+\sum_{k=1}^{N}a_{ik}x_{k2}\right\}\text{,}$$

where

(Equation 5) $${\hat{r}}_{i2}\left({\vec{x}}_{1}\right)\equiv r_{i2}+\sum_{k=1}^{N}a_{ik}x_{k1}$$

and ${\vec{x}}_{1}=\left(x_{11},x_{21},...,x_{N1}\right)$. In other words, the realized intrinsic growth rate of species i in the downstream patch ${\hat{r}}_{i2}$ is decomposed into its baseline $r_{i2}$ (given by the growth rate when no species exist in the upstream patch) and the effects of the upstream community $\sum_{k=1}^{N}a_{ik}x_{k1}$. As the downstream species cannot affect the upstream dynamics, changes in the upstream community can be seen as environmental changes from the perspective of the downstream communities. We use Sp(i,T) to represent whether the invasion of species i in the upstream community drives one or more downstream species at time step T extinct or not:

(Equation 6) $$\text{Sp}\left(i,T\right)=\left\{ \begin{array}{cc} 0 & \left(\text{extinction}\right)\\ 1 & \left(\text{otherwise}\right) \end{array}\right.$$

We emphasize that structural persistence Sp differs from structural stability,^39^^,^^40^^,^^41^^,^^42^^,^^43^^,^^44^^,^^45^ although both consider a perturbation in ${\vec{\hat{r}}}_{2}$. Structural stability considers the sudden jump of ${\vec{\hat{r}}}_{2}\to \;{\vec{\hat{r}}}_{2}+\vec{\Delta }$ where $\vec{\Delta }$ represents the changes in growth rates. On the other hand, resistance to environmental change here indicates the gradual change in ${\vec{\hat{r}}}_{2}$ because such changes are caused by the dynamics of the upstream community.

#### Species migration

To calculate the stability, we need to know the probability that each species invades each patch. We consider three types of migration: (i) from outside the meta-community (hereafter, called the migration pool) to the upstream patch, (ii) from the migration pool to the downstream patch, and (iii) from upstream to downstream patch (Figure 2A). We emphasize that an invader should be a member of the upstream community in migration type (iii) while such membership is not necessary in migration type (ii). In all migration types, the abundances of invader species are fixed at 0.01 at t=0.

The probabilities of species migration into each patch can be written as follows:

(Equation 7a) $$p_{i1}=\frac{\rho }{N+\mu \sum_{i=1}^{N}\delta \left(x_{i1}\right)}$$

(Equation 7b) $$p_{i2}=\frac{\left(1-\rho \right)+\mu \delta \left(x_{i1}\right)}{N+\mu \sum_{i=1}^{N}\delta \left(x_{i1}\right)}\text{,}$$

where $p_{ij}$ represents the probability of species i’s migration to patch j, ρ and 1−ρ represent the frequencies of migration events from the migration pool to the upstream and downstream patches (0≤ρ≤1), respectively, μ≥0 scales the frequencies of migration events of species i from the upstream to downstream patch, and $\delta \left(x_{i1}\right)$ is a function to give the frequency of species i’s migration from the upstream to the downstream patch, depending on the abundance of the focal species in the upstream patch. In this manuscript, we consider the simplest form of this function: upstream species have identical migration frequencies from the upstream to the downstream patch regardless of their abundances. The form of δ in this manuscript is written as follows:

(Equation 8) $$\delta \left(x\right)=\left\{ \begin{array}{cc} =1 & \left(x>0\right)\\ =0 & \left(x\leq 0\right)\text{.} \end{array}\right.$$

This form of δ clarifies the interpretation of μ’s effect: we can scale the migration from up- to downstream against the migration from the migration pool. If δ has another functional form, the interpretation of μ gets more complicated.

Some parameter sets of (ρ,μ) correspond to intuitive scenarios of meta-community dynamics. For example, (ρ,μ)=(1,0) and ρ=0 are the cases where species migrate only to upstream and downstream patches, respectively. (ρ,μ)=(0.5,0) represents the mainland-island model,^59^ except that the upstream community affects the downstream dynamics. ρ=1 with μ>0 represents the cases where the migration is perfectly hierarchical: migration only occurs from the migration pool to the upstream patch and from the upstream to the downstream, although μ changes which types of migration are more likely to occur.

The downstream stability at time point T (or $q_{0}\left(T+1\right)$) is then written as follows:

(Equation 9) $$\begin{array}{cc} q_{0}\left(T+1\right) & =1-\sum_{i\neq 0}q_{i}\left(T+1\right)\\ & =1-\sum_{i=1}^{N}p_{i1}\left(1-\text{Sp}\left(i,T\right)\right)-\sum_{i=1}^{N}p_{i2}\left(1-\text{Ri}\left(i,T\right)\right)\\ & =\sum_{i=1}^{N}\left(p_{i1}\text{Sp}\left(i,T\right)+p_{i2}\text{Ri}\left(i,T\right)\right)\text{.} \end{array}$$

This equation clarifies that the migration parameters, ρ and μ, weight the effects of the two types of resistance on downstream stability. Equation 9 also indicates that our system is Markovian: The stability at time T depends only on species abundances and migration rates at T. We do not care about the history of meta-community dynamics and we can easily extend the model to include time-varying migration rates. In addition, we can measure stability from only 2N invasion simulations at each time T: we need to run a simulation where species i (i=1,...,N) invades either upstream or downstream. Note that our measure of stability is entirely deterministic.

#### Two sampling scenarios

We analyzed the stability of downstream communities in two scenarios. For each scenario, we generated 60 sets of N=25 species (migration pool) with different interaction matrices A, and growth rates ${\vec{r}}_{1}$, and ${\vec{r}}_{2}$, and we varied the migration parameters for each set. See Data S1.3 for more details.

In the first, the assembly scenario (Figure 2C), we simulate how a community might assemble in nature: starting from two empty patches, we simulate 250 migration events (T=1,2,...,250) given the values of $A=\left(a_{ik}\right)$, ${\vec{r}}_{1}=\left(r_{11},...,r_{N1}\right)$, ${\vec{r}}_{2}=\left(r_{21},...,r_{N2}\right)$, ρ, and μ. We then measure the longevity of downstream species composition and sample many meta-communities. However, this method does not randomly sample meta-communities as the sampled upstream and downstream communities depend on them at the previous time step; the statistical analysis for this sampling method would be very difficult.

The second is the design scenario (Figure 2D), which represents how one might want to design a target community with known properties: we define 60 target downstream communities composed of species randomly sampled from N species; species richness is uniformly chosen from 1 to 10 because species richness is expected to reduce coexistence when species interactions are random^49^ and larger stable communities were very difficult to generate. These target communities are feasible and locally stable in the absence of upstream communities. We calculated the stability of each target downstream community over randomly generated 200 upstream communities. In this scenario, we assume that we know the downstream species composition that is the most beneficial to the host and we want to stabilize this composition. Then, we can ask what designs of upstream communities increase downstream stability. In contrast to the assembly scenario, we randomly sample the upstream and downstream communities in the design scenario: we can use these data in regression analysis. However, as some upstream communities collapse the target downstream communities, this sampling method is not effective to collect data (Figure S3). Although it is difficult to derive the general conditions where the upstream community does not collapse the target downstream species composition, we can derive what kinds of upstream communities would not collapse a target downstream community when all interactions in the downstream community are negative (Figure S9) by using the results of previous studies.^43^^,^^60^ See Data S3 for more details.

#### Manipulating positive interactions

Manipulating positive interactions between certain species can change community features other than the total strength of positive interactions from the upstream to the downstream communitpacsies. To avoid this problem, we generated meta-communities where species compositions in the upstream and the downstream communities do not overlap (e.g., species 1– 8 were in the upstream patch while species 9 – 16 were in the downstream patch). To generate such meta-communities, we first generated 11 eight-species communities where species can coexist in the absence of spatial structure. The coexistence was tested given two growth vectors, which represent the species’ growth vector in the upstream patch and downstream patch, respectively. This means that these eight species should coexist (i) in the upstream patch and (ii) in the downstream patch when no species interactions between the communities exist. The growth rates and the species interaction matrices were sampled as explained in Data S1.3.

Then, we allocated one of the 11 eight-species communities in the upstream patch and another to the downstream patch (in total, we have 11×10=110 meta-communities). As we assumed that species compositions did not overlap, we can manipulate the species interactions between communities without changing species interactions within upstream and downstream communities, respectively. To clarify the effect of positive interactions from upstream to downstream communities, we assumed that all interactions from the upstream species to the downstream species were positive. On the other hand, we assumed that all interactions from the downstream species to the upstream species were negative, which makes the mean of the off-diagonal elements of the interaction matrix zero. To do so, we sampled species interactions between communities from modified half-normal probability distributions. In short, a species interaction matrix is given by the following 16×16 block matrix:

(Equation 10) $$\left(\begin{array}{cc} A_{up} & B^{-}\\ B^{+} & A_{down} \end{array}\right)$$

where $A_{\text{up}}$ and $A_{\text{down}}$ represents species interactions within the upstream and the downstream patches, respectively, while $B^{\pm }$ represents interactions from the upstream species to the downstream species and vice versa. (i,k) element of either $A_{\text{up}}$ or $A_{\text{down}}$ (we use $a_{ik}$ for simplicity) is give as below:

(Equation 11) $$a_{ik}\left\{ \begin{array}{ll} =-1 & \text{if}\;i=k\\ ∼N\left(0,0.25^{2}\right) & \text{otherwise.} \end{array}\right.$$

On the other hand, (i,k) element of matrix $B^{\pm }$, $b_{ik}^{\pm }$, follows the following probability distribution with parameter σ:

(Equation 12) $$b_{ik}^{\pm }∼\pm \frac{h\left(y\right)}{\sigma }$$

where

(Equation 13) $$y=\frac{b_{ik}^{\pm }}{\sigma }-1$$

(Equation 14) $$h\left(y\right)=\sqrt{\frac{2}{\pi }}\exp\left(\frac{-y^{2}}{2}\right)\;\left(\text{half}-\text{normal}\;\text{distribution}\right)\text{.}$$

We manipulated the total strength of positive interactions from the upstream community to the downstream community by charging σ.

We fixed the migration parameter values as (ρ,μ)=(0.5,0.5) to exclude the effects of these parameters on stability.

#### Pseudo-spatially structured model

In the analysis of pseudo-spatially structured model, we analyzed a special case of community dynamics without the spatial structure. Here, we have species 0 that is not affected by the other species but affect the growth of them. To generate an interaction matrix in this scenario, we first generated 24×24 matrix A following Equation S7 for interactions among species i=1,...,24. The interactions with species 0 is given by

(Equation 15a) $$a_{00}=-1$$

(Equation 15b) $$\begin{array}{cc} a_{0i}∼N\left(0,0.25^{2}\right) & i=1,...,24 \end{array}$$

(Equation 15c) $$a_{i0}∼\left\{ \begin{array}{ll} \text{sign}\left(m\right)\text{Beta}\left(10\left|m\right|,10\left(1-\left|m\right|\right)\right) & \text{if}\;\text{species}\;i\;\text{is}\;a\;\text{resident}\;\text{species}\\ N\left(0,0.25^{2}\right) & \text{otherwise} \end{array}\right.$$

where m gives the mean strength of interactions from species 0 to chosen resident species (m=±0.1,±0.3,±0.6,±0.9), sign(m)=1 (if m>0) or −1 (otherwise), and Beta(α,β) represents the beta distribution with two parameters α and β which determine the mean and the variance. We generated 30 interaction matrices for each value of m.

In this simulation, we first choose three species from i=1,...,24 that coexist with species 0, and measured the stability of these communities. Then, we removed species 0 and asked whether the three resident species coexist or not. If they cannot coexist, we excluded such communities. If the three resident species coexisted, we measured the stability while not allowing the invasion of species 0. As this manipulation changes the number of species in the migration pool (25 species with species 0, and 24 species without species 0), we multiplied the stability without species 0 by 25/24 when we compare the stability with and without species 0. Finally, we performed the Spearman correlation test between the total strength of positive or negative interactions from species 0 to the resident species and the difference in stability with species 0 minus without species 0.

### Qualification and statistical analysis

#### Causal inference

After calculating the stability of the downstream communities, we statistically analyzed how features of communities (Table 1) relate to the stability of the downstream communities. As the stability is the probability that the downstream composition is maintained, we performed the logistic regression analysis using logit function in statsmodels.formula.api.^61^ The community features were calculated from realized species interactions within or between the two patches, the number of species in each patch, and the two migration parameters (Table 1). These features were standardized so that means are 0s and the standard deviations are 1s using preprocessing.StandardScaler.fit().transform() function in package scikit-learn.^62^ This enabled us to compare the effects of each feature without considering the difference in their scales.

In the main text, we analyzed the causal effects of the following five features related to the upstream communities: species richness in the upstream, total strength of positive or negative interactions within the upstream community, and total strength of positive or negative interactions from the upstream to the downstream. The rationale of this analysis is as follows: assuming that we want to stabilize a certain downstream community that is beneficial to a host but the manipulation of the downstream community is difficult, we may be able to change the stability of the downstream community by manipulating the upstream community. For the causal inference in the absence of the spatial structure or the effects of other features (e.g., total strength of interactions in the downstream patch), see Data S2 and S6, respectively.

To perform the causal inference,^46^^,^^47^^,^^48^ we assumed the causal relationships between the community features and the stability metrics represented by Figure 3. First, we can expect that species richness increases (or, at least, does not decrease) the total strength of positive/negative interactions as well as the mean degree within upstream and downstream communities, respectively, because the number of species interactions increases with the number of species. Species richness in both communities should increase the total strength of positive/negative interactions and a degree from the upstream community to the downstream community for the same reason. According to the previous studies,^35^^,^^36^^,^^37^ species richness increases the resistance to invasion because of resource competition. Although our model (Equations 1a and 1b) does not explicitly include resource competition, species interaction in the model can relate to resource competition (i.e., positive interactions: providing resources, and negative interactions: competing for resources). Then, we can expect that the total strength of interactions affects the resistance to invasion within upstream and downstream communities, respectively. To our best knowledge, there is no study that suggests the causal relationship between the degrees and the two types of resistance, and Equations 1a and 1b do not include the degrees. For these reasons, we do not consider the causal relationships from the degrees to the stability metrics, although relaxing this assumption does not affect the conclusion, see Table S11. We do not consider the causal effects of mean strength of positive or negative interactions on the stability metrics for similar reasons. The resistance to invasion in *the upstream community* would increase the resistance to the environmental change because the downstream environment would not change if no species can colonize the upstream community. In addition, we can expect that the total strength of positive and negative interactions from the upstream to the downstream communities affect the resistance to the environmental change and resistance to invasion: the strong interactions indicate that the changes in the upstream community propagate the downstream dynamics, and the interactions from the upstream to the downstream affect the growth rates of invader species in the downstream community (see Equation 5). Finally, the two migration parameters ρ and μ affect the resistance to the environmental change and resistance to invasion as these migration parameters determine whether species are more frequently invade the upstream communities or the downstream communities. We changed which features should be included in the logistic regression depending on whose effects we want to estimate (Table S9) to satisfy the backdoor criterion^47^ in the causal diagram of Figure 3.

#### Statistical tests

In this manuscript, we performed Wilcoxon rank-sum test, Wilcoxon signed-rank test, and Spearman correlation analysis using scipy version 1.6.2^58^ and pingouin version 0.5.3^63^ with Python version 3.8.8. To report effect sizes, we used the rank-biserial correlation in the singed-rank tests, and Cliff’s delta from cliffs_delta library^64^ in the rank sum tests, respectively. These two non-parametric effect sizes reflect how many samples from one group are larger or smaller than samples from the other group; they produce values between −1 (all values of the second sample are larger than all the values of the first sample) and 1 (vice versa). Cliff’s delta can be used to interpret effect sizes as small (±0.11≶), medium (±0.28≶), and large (±0.43≶),^65^ but this interpretation should not be used rigidly.

#### Programming

All mathematical and statistical analyses were performed in Python (version 3.8.8) with the following packages: numpy version 1.20.1,^66^ scipy version 1.10.0,^58^ statsmodels version 0.13.1,^61^ and scikit-learn version 0.24.1.^62^

## Data Availability

• Data: Simulation data in csv files are available on Zenodo (key resources table). • Codes: Python codes in Jupyter Notebooks are available on Zenodo (key resources table). • The simulation codes and data used in this study are available on Zenodo https://doi.org/10.5281/zenodo.7959283

## Supporting citations

The following references appear in the Supplemental Information: 67,68,69.
