# Supplementary material for: A spatially structured mathematical model of the gut microbiome reveals factors that increase community stability
Source: iScience. 2023 Jul 31;26(9):107499. doi: 10.1016/j.isci.2023.107499 (PMC10475486; doi:10.1016/j.isci.2023.107499)
Supplement: Document S1. Data S1–S8, Figures S1–S15, and Tables S1–S9 [file mmc1.pdf]

iScience, Volume *26*

Supplemental information

A spatially structured mathematical model of the gut microbiome reveals factors that increase community stability

Shota Shibasaki and Sara Mitri

Data S1 The details of the model, related to the STAR Methods

Data S1.1 Derivation

In the main text, we use a generalized Lotka-Volterra (gLV) model. This model can describe the community dynamics with a small number of parameters but gLV models may not be easy to biologically interpret, especially for non-theoretical biologists. In this section, we explain the derivation of the model so that our results could be easily tested with empirical data.

First, we consider the following Lotka-Volterra model, which would be easier to fit the experimental data than Eqs (1a) and (1b):

$$\frac{dx_{i1}}{dt} = \mu_{i1}x_{i1}\left(K_{i1} + \sum_{k=1}^{N} A_{ik}x_{k1}\right) \tag{S1a}$$

$$\frac{dx_{i2}}{dt} = \mu_{i2}x_{i2}\left\{K_{i2} + \sum_{k=1}^{N} A_{ik}(x_{k1} + x_{k2})\right\} \tag{S1b}$$

where K_{ij} is the carrying capacity of species i in patch j in mono-culture, $\mu_{ij}K_{ij}$ represents the intrinsic growth rate in mono-culture of species i in patch j,and A_{ik} is the species interaction from species k to i with $A_{ii} = -1$ for all i. Depending on the scales of microbial abundances, one may use the log of the abundances, which changes estimated parameter values. However, using log-scale does not change the signs of parameters or the following procedures.

The above ordinary differential equations are rewritten as follow

$$\frac{dx_{i1}}{dt} = x_{i1}\left(r_{i1} + \sum_{k=1}^{N} \hat{a}_{ik}^{(1)}x_{k1}\right) \tag{S2a}$$

$$\frac{dx_{i2}}{dt} = x_{i2}\left\{r_{i2} + \sum_{k=1}^{N} \hat{a}_{ik}^{(2)}(x_{k1} + x_{k2})\right\} \tag{S2b}$$

where

$$r_{ij} = \mu_{ij}K_{ij}, \tag{S3}$$

$$\hat{a}_{ik}^{(j)} = \mu_{ij}A_{ik} \tag{S4}$$

As the main text assumes $\vec{r}_1 \neq \vec{r}_2$ and $a_{ik}^{(1)} = a_{ik}^{(2)}$, the patches are assumed to change K_{ij} but not μ_{ij}. If the growth parameter μ_{ij} differs over the patches, we need to reformulate Eqs (1a) and (1b) using $\hat{a}_{ik}^{(1)}$ and $\hat{a}_{ik}^{(2)}$ instead of a_{ik}. In addition, as we assume $a_{ii} = -1$, we considered the special case of $\mu_{ij} = 1$ in the main text.

Data S1.2 Convergence of the dynamics

In the main text, we run the dynamics of Eqs (1a) and (1b) until $t = 300$ for every invasion event. To test whether $t = 300$ is long enough, we compared the population abundances at $t = 300$ and $t = 600$. We measured the difference between the two-time points by calculating the scaled mean difference of all species in the two

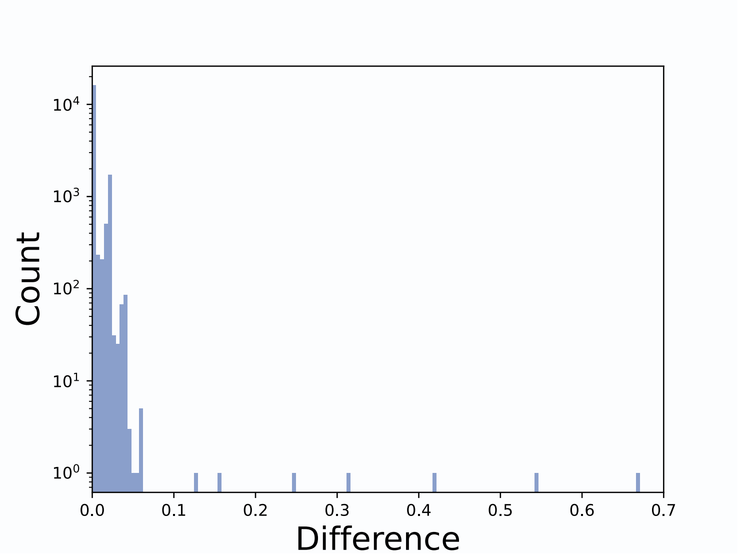

Figure S1: Convergence confirmation, related to STAR Methods

The distribution of scaled mean difference of population abundances between $t = 300$ and $t = 600$ in the design scenario with the spatial structure. For example, a Difference $= 0.05$ indicates that the average population abundance $x_{ij}(600)$ is 5% larger or smaller than $x_{ij}(300)$ on average (across i and j).

patches:

$$\text{Difference} \equiv \frac{1}{2N} \sum_{i=1,2} \sum_{j=1}^{N} \frac{|x_{ij}(300) - x_{ij}(600)|}{x_{ij}(300)}. \tag{S5}$$

If the dynamics converge very slowly and $x_{ij}(600) = 2x_{ij}(300)$, for example, Difference $= 1$. If $t = 300$ is long enough to stabilize the dynamics, the above difference should remain close to zero, as we see in Fig. S1 for the design scenario with spatial structure.

Data S1.3 Sampling parameter values

To see the relationship between the stability and the features of the upstream and downstream communities, we sampled the parameter value from the following probability distributions

$$r_{ij} \sim U(0, 2) \tag{S6}$$

$$a_{ik} \begin{cases} = -1 & \text{for } i = k \\ \sim \mathcal{N}(0, 0.25^2) & \text{with probability } C \text{ for } i \neq k \\ = 0 & \text{otherwise} \end{cases} \tag{S7}$$

where $U(a, b)$ represents the uniform distribution from a to b, $\mathcal{N}(\mu, \sigma^2)$ represents the normal distribution whose mean and standard deviation are μ and σ, respectively, and C represents the connectance of species interaction matrix. We assumed that all species can grow in mono-culture ($r_{ij} > 0$) and that the strength of species interactions is unlikely to be stronger than that of intraspecific interactions ($\sigma = 0.25$). We set $C = 0.25, 0.5, 1.0$ and sampled 20 sets of $\vec{r}_1$, $\vec{r}_2$, and a_{ik} for each C.

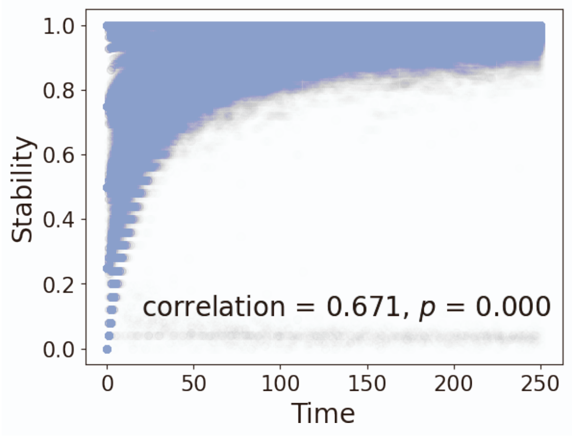

Figure S2: Stability in the assembly scenario over time, related to Figure 2

Stability of the downstream communities in the assembly scenario over time. Spearman correlation coefficient: 0.671 and $p < 10^{-3}$.

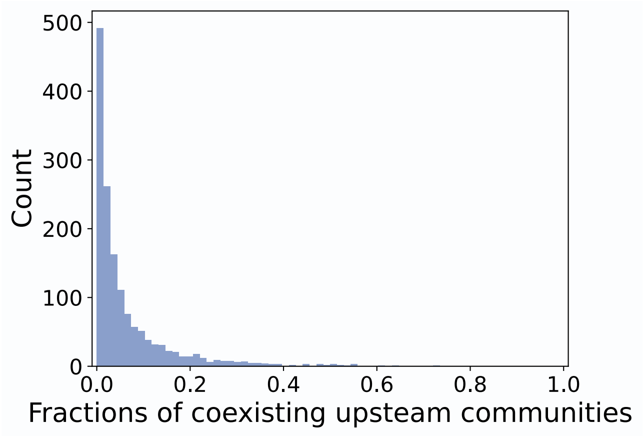

Figure S3: Fractions of upstream communities coexisting with the downstream, related to Figure 2

Fractions of upstream communities coexisting with the target downstream communities. For each of 1,500 target downstream communities, we randomly generated 200 upstream communities.

For each set of parameters related to the species interactions and the growth rates, we calculated the stability in the two scenarios – assembly and design – over the parameters related to migration: $\rho = 0.0, 0.25, 0.5, 0.75, 1.0$ and $\mu = 0.0, 0.25, 0.5, 1.0, 2.0$. In the stability analysis of the assembly scenario, the downstream stability positively correlates with the time steps the meta-communities were sampled (Fig. S2, Spearman correlation: 0.671 and $p < 10^{-3}$). In the design scenario, the majority of sampled upstream communities cannot coexist with the target downstream communities: one or more species go extinct in either or both of the upstream and downstream communities before the dynamics stabilized (Fig. S3). See Data S3 for the discussion of the coexistence in the meta-communities.

Data S2 Statistical analysis without the spatial structure, related to Figure 1

As a control scenario, we analyzed the cases where we have no spatial structure in this section. We have only one community in this case. The results in this section can be regarded as the stability without spatial structure as well as that in the upstream community in the structured scenario. This is because the spatial structure does not affect the migration probability to the upstream or the community dynamics there.

We generated communities in the absence of the spatial structure using the target downstream communities in the designing scenario. Now, the species always migrate from the outside of the community and thus the stability only represents the resistance to invasion. In this case, species richness has the largest positive correlation with the stability (Fig. S4), which support the findings in the previous studies[34;35;36;37].

We also performed the causal inference in this scenario. We assumed the causal relation similar to the main text: species richness increases the total strength of positive and negative interactions, respectively, and these interactions relate to the stability. To satisfy the backdoor criteria, the logistic regression model includes only the richness to see the effect of richness, while we included richness, the total strength of positive interactions, and the total strength of negative interactions to the see effects of the total strength of positive and negative interactions, respectively, on the stability. Table S1 shows that only species richness has a significant effect on the stability. This explains why communities in the assembly scenario are more stable than in the design scenario without spatial structure. First, Fig. S5 shows that species richness in the assembly scenario exceeds that in the design scenario (from one to ten). If we sub-sample communities whose species richness is ten or less from the assembly scenario, the difference of stability between the two scenarios decreases (Fig. S6, Wilcoxon rank-sum test: $U = -4.867$, $p = 1.13 \times 10^{-6}$, Cliff's delta is -0.377), but the designed communities are still less stable than the assembled ones. This may be because species richness in the design scenario is still lower than the sub-sampled assembly scenario (Fig. S7, Wilcoxon rank-sum test: $U = -3.651$, $p = 2.62 \times 10^{-4}$, Cliff's delta is -0.283). With spatial structure, sub-sampling the assembly scenario has little effect on downstream community stability (Fig. S8), suggesting a smaller role for richness in the spatially structured model.

The above results can be used to consider the upstream stability in the spatial structure we considered in the main text. As the upstream patch is not affected by the spatial structure, we can infer that the upstream stability is high when the upstream richness is large.

Table S1: Causal inference without spatial structure, related to Figure 1

Feature	Coefficient	Standard error	P-value
Richness	0.2813	0.128	0.028
Total positive	0.1357	0.293	0.643
Total negative	−0.1733	0.302	0.567

In the main text, we analyzed the downstream stability while the result of this section infer the upstream stability. If we need to consider the stability of both the upstream and downstream communities (i.e., meta-community stability), we would just modify the definition of the stability so that we consider the resistance

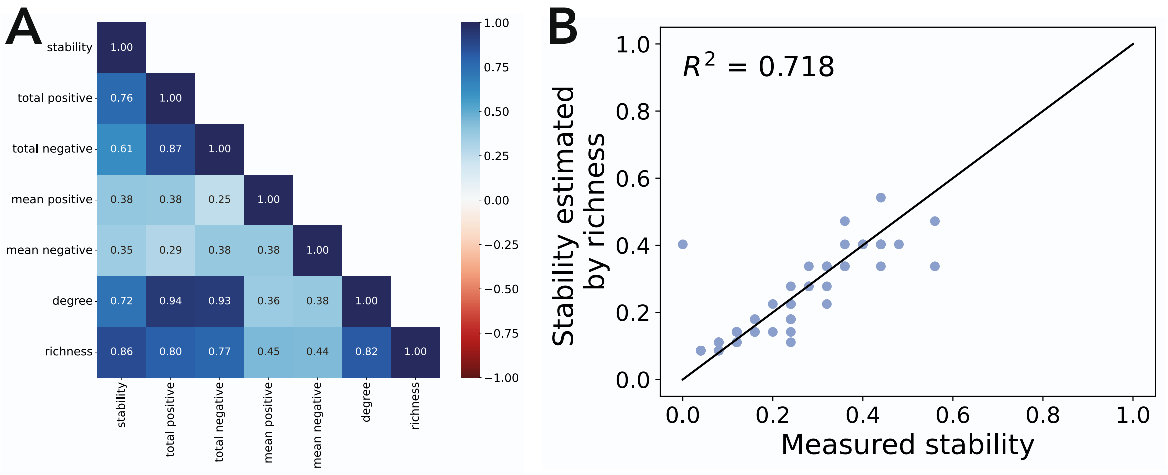

Figure S4: Analysis without spatial structure, related to Figure 1

A: Pearson correlations in the absence of the spatial structure. Darker blue represents stronger positive correlations while darker red indicates stronger negative correlations. Stability (resistance to invasion) has the largest positive correlation with species richness. B: comparison of the measured stability with the prediction using a logistic regression model with species richness.

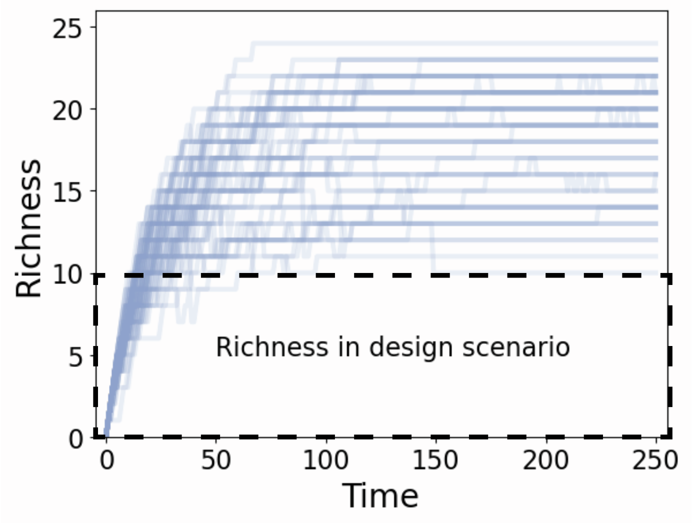

Figure S5: Species richness in the assembly scenario, related to Figure 1

Each blue line represents how species richness in a community changes over time in the assembly scenario without the spatial structure. The black dashed line represents the range of species richness (from one to ten) in the design scenario without the spatial structure.

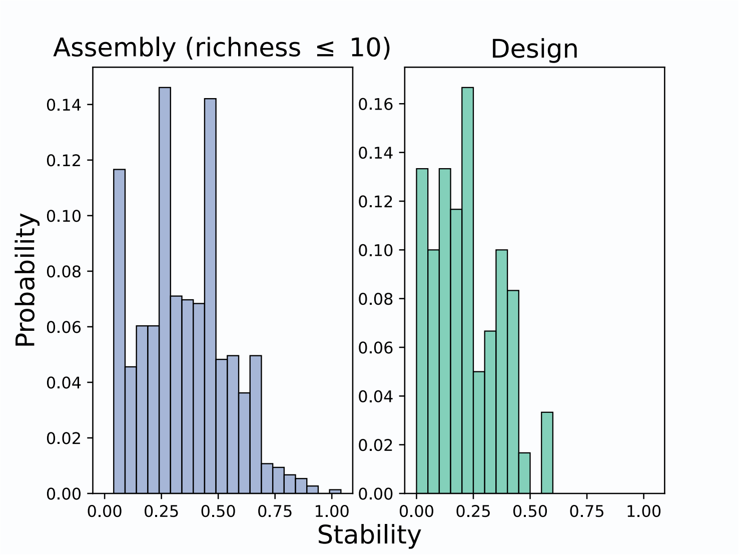

Figure S6: Distributions of downstream community stability without spatial structure, related to Figure 1

Probability distributions of downstream community stability in the sub-sampled assembly scenario (left) and the design scenario (right) without spatial structure are shown.

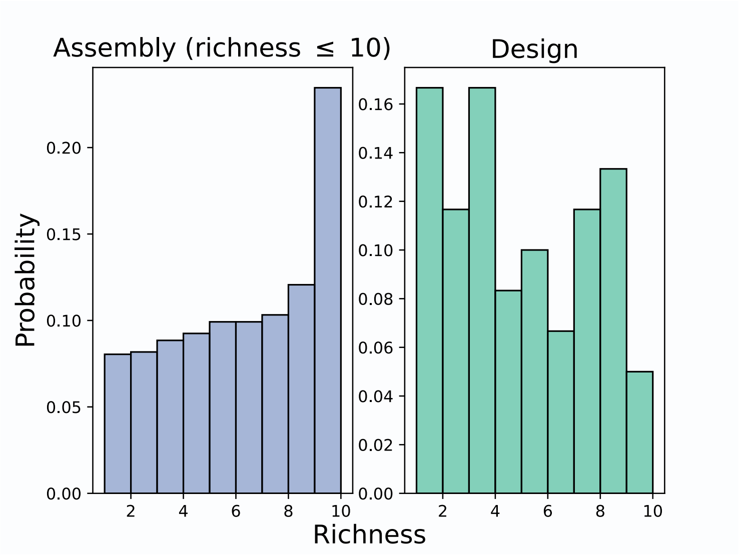

Figure S7: Distributions of species richness without spatial structure, related to Figure 1

Probability Distributions of species richness in the sub-sampled assembly scenario (left) and the design scenario (right) without spatial structure are shown.

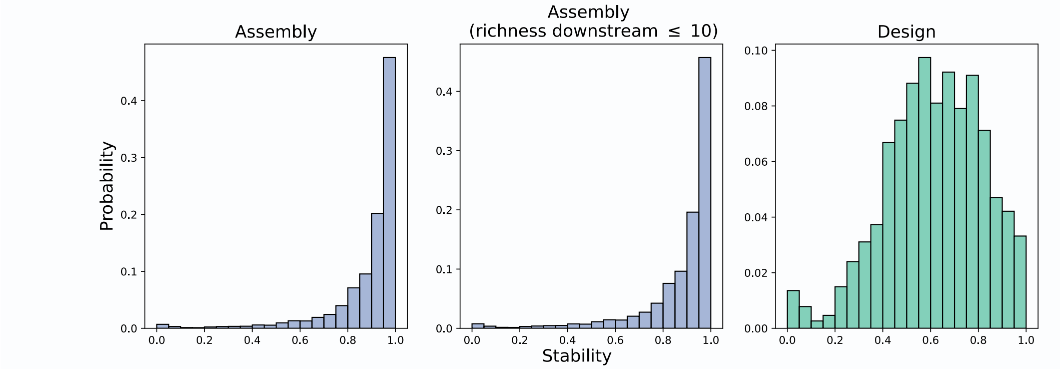

Figure S8: Distributions of downstream community stability with spatial structure, related to Figure 2

Probability Distributions of downstream community stability in the full (left), sub-sampled assembly scenario (center, richness downstream ≤ 10) and the design scenario (right, richness downstream ≤ 10) with spatial structure are shown.

to invasion in the upstream and downstream communities. We do not have to consider the resistance to the environmental changes caused by upstream communities in such cases because the environmental changes do not occur unless invaders colonize the upstream community. In this sense, the downstream stability in the main text can be seen as an upper boundary of the meta-community stability.

Data S3 Coexistence conditions, related to Figure 2

In the designing scenario, we analyzed the meta-communities where (i) the target downstream communities are feasible and locally stable in the absence of the upstream community, (ii) the accompanying upstream communities are also feasible and locally stable, and (iii) the upstream communities do not drive one or more species extinction in the downstream. Such meta-communities should satisfy the following three necessary conditions

- Hierarchy: because the downstream dynamics stabilize after the stabilization of the upstream dynamics, all species in the downstream should have positive abundances until then. In other words, the upstream community should stabilize enough fast so that no species in the downstream goes extinct.
- Feasibility: after the upstream community stabilizes, we should check whether the downstream dynamics have an equilibrium where all downstream species can coexist. As the upstream species affect the growth rates of the downstream species, such feasible equilibrium may not exist in some cases.
- Convergence: if the downstream community has a feasible equilibrium in the presence of the upstream community, we need to know whether the downstream dynamics converge to the feasible equilibrium. In this case, the local stability may not be sufficient, because the effects from the upstream community can drive the downstream community far from the equilibrium. If the feasible equilibrium is globally stable, on the other hand, the downstream community converges to the equilibrium.

Analyzing these three conditions, in general, would be very difficult. However, if we assume that the initial condition of the upstream community is very close to the equilibrium, we can omit the first condition. In

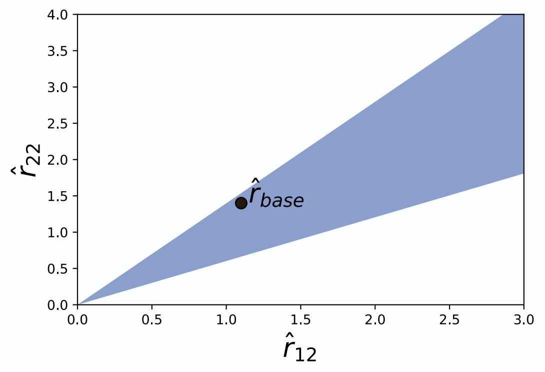

Figure S9: Region of allowing two competitive species coexistence, related to Figure 2

The colored area represents the region of $(\hat{r}_{12}, \hat{r}_{22})$ that enable two competing species to coexist in the downstream patch. The black dot represents the growth rates of the two species when no species exist in the upstream patch. The upstream community changes the two species' growth rates $(\hat{r}_{12}, \hat{r}_{22})$ from the basal growth rates. Parameter values: $a_{11} = -1$, $a_{12} = -0.715$, $a_{21} = -0.603$, $a_{22} = -1$, $\hat{r}_{12} = 1.1$, and $\hat{r}_{22} = 1.4$. Therefore, this example indicates that species 1 grows slower and less competitive than specie 2.

addition, we can analyze the second and third conditions (feasibility and convergence) by assuming that all species interactions in the downstream community are negative (i.e., a competitive community).

Let us start with a simple downstream: we have only two species there and they are competitive ($a_{ik} < 0$ for $i = 1, 2$ and $k = 1, 2$). Assuming that the upstream community is stabilized, we consider the generalized Lotka-Volterra equation given by Eq (4). It is widely known that the feasible equilibrium is globally stable in this classical example if and only if[43;67]

$$\underbrace{\sqrt{\frac{a_{11}a_{22}}{a_{12}a_{21}}}}_{\text{Niche overlap}^{-1}} > \underbrace{\frac{\hat{r}_{12}}{\hat{r}_{22}}\sqrt{\frac{a_{22}a_{21}}{a_{11}a_{12}}}}_{\text{fitness difference}} > \underbrace{\sqrt{\frac{a_{12}a_{21}}{a_{11}a_{22}}}}_{\text{Niche overlap}}, \tag{S8}$$

$\hat{r}_{21}, \hat{r}_{22} > 0$, and $0 < a_{12}a_{21} < a_{11}a_{22}$. In the modern coexistence theory[60], inequalities (S8) are understood by niche overlap and fitness difference. Recall that the effects from upstream community is summarized in $\hat{r}_{12}$ and $\hat{r}_{22}$, see Eq (5). Then, the upstream community only changes the fitness difference and we can derive the conditions where the upstream community enables the two species coexist in the downstream (Fig S9).

If we have three or more species in the downstream patch and there are only negative interactions in the downstream community, we can use the structural approach suggested by Saavedra *et al*[43]. Again the upstream community's effects appear in the fitness difference but not in the niche overlap. Importantly, the fitness difference and the niche overlap defined by Saavedra *et al*[43] show only the feasible condition. The sufficient condition for the global stability of the equilibrium is that the interaction matrix in the downstream community is Volterra-dissipative. See Appendix 3 of Saavedra *et al*[43] for more mathematical details.

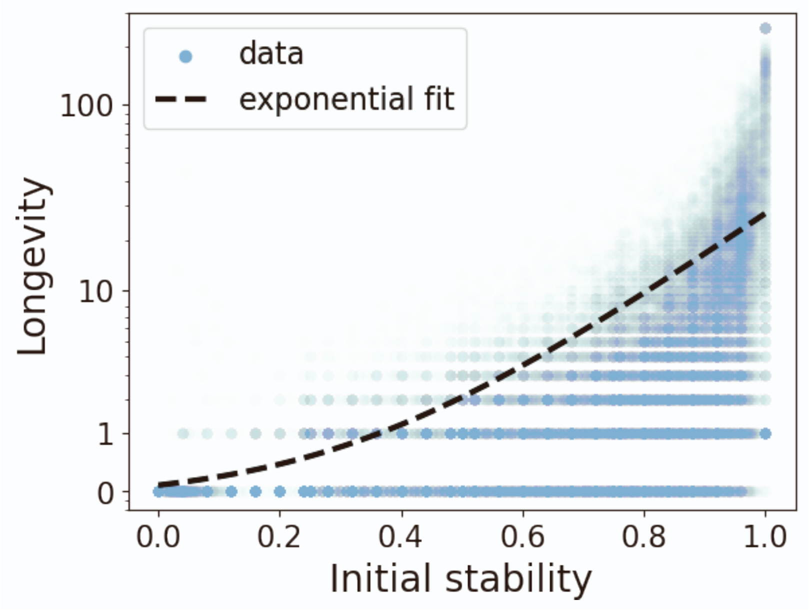

Figure S10: Stability explains the longevity of species compositions, related to Figure 2

Each blue dot represents the downstream community in the assembly scenarios ($n = 37998$). The initial stability of the downstream community depends on the downstream community as well as the accompanying upstream community when the focal downstream community is composed. Then, we calculated the duration that the downstream species composition is maintained. The black dashed line represents the fitting to the following exponential function: longevity $= 1.780 \times 10^{-1} \times \exp(5 \times \text{initial stability}) + 5.141 \times 10^{-16}$. The coefficients of this function were obtained by scipy.optimize.curve_fit.

Data S4 Longevity of downstream communities, related to Figure 2

The data from assembly scenario were used to measure how long a focal downstream species composition is maintained (Fig. S10). As discussed in the main text, the downstream species composition depends on upstream and downstream communities: the downstream stability can change when it's upstream community changes the species composition. However, if the initial stability (the stability that the focal downstream species composition is generated) is large, the focal downstream species composition is maintained.

Data S5 Prediction analysis, related to Figure 3

In this section, we build some logistic regression models to predict the stability because measuring the stability in experiments is difficult. Here, we used the data in the desig scenario to train the statistical models because the assembly scenario does not randomly sample the meta-communities. However, we used sub-sampled data of the assembled to see the performance of the statistical prediction. As the stability in the assembly scenario is biased, we sub-sampled 100 data of stability $[0.1 \times i, 0.1 \times (i + 1)]$ fro $i = 0, \ldots, 9$.

First, we justify the choices of community features. We analyzed species richness in the upstream and downstream patches, respectively, because the previous studies suggest that species richness affects the resistance to invasion[34;35;36;37], which is also supported by our analysis without the spatial structure (Data S2). In addition,

because the resistance to the environmental changes is conceptually similar to structural stability, we measured the total strength of positive or negative interactions and the mean degree (i.e., how many species each species interact with), which can correlate with structural stability[68]. We, therefore, evaluated the total strength of positive or negative interspecific interactions within the upstream and downstream communities, respectively, the total strength of positive or negative interactions from the upstream to the downstream communities including intraspecific interactions, mean degree within and upstream and downstream communities, respectively, and mean degree between the two communities. We also quantified the mean strength of positive or negative interaction within each patch, and from the upstream to the downstream, respectively. The explanations of features are summarized in Table 1. We emphasize that the models in this section include features that are ignored in the causal inference. This is because certain features may be useful to predict the stability because of correlation, although they are unlikely to have causal effects on the stability.

After calculating these 17 community features, we investigated the best logistic regression model to predict the downstream community's stability using the community features and the two migration parameter values (ρ and μ). The brute force search for the best predictive model is, however, not feasible because we have to analyze $2^{19} - 1 \approx 500,000$ logistic regression models. Instead, we generated some models based on the step-wise algorithms. First, we considered the null model: i.e., the model has only the intercept term (Table S3). This model can be seen as a baseline. Second, we generated a logistic regression model using the forward selection: from the null model, we introduced a single feature into the model that increased McFaddens pseudo-R squared the most and repeated this process until the pseudo-R squared did not increase. We call this model the forward model 1 in this section (Table S4). In addition, we can consider another baseline using the full model1, which has all 19 features and the intercept (Table S5). Then we performed backward selection: we removed each feature from the full model 1 that had the highest p-value and repeated this process until all remaining features had p-values smaller than 0.05. We call this logistic regression model the backward model (Table S2). Then, we calculated the variance inflating factors (VIFs) of the three models – the full model1, the forward model1, and the backward model – using the statsmodels package because VIFs > 5 can cause collinearity[69]. Indeed, the full model and the forward model1 have large VIFs while the backward model has small VIFs. We generated additional two models, the full model 2 (Table S6) and the forward model 2 (Table S7), by removing features that have the highest VIF in the full model 1 and the forward model 1, respectively, and continued removing features with the largest VIF until all features in the model had VIFs less than 5.

Within these six logistic regression models – the null model, the full model 1, the full model 2, the forward model 1, the forward model 2, and the backward model– we chose the best predictive model based on Akaike information criteria (AIC). AIC suggested that the backward model is the best model to predict the stability (Table S8). Fig. S11 visualizes the comparisons of the true stability with the predicted stability by the backward model using the data from the designing scenario (A) and the assembly scenario (B). Because we have a small number of data with stability < 0.4, the model cannot predict such small stability. For the other models. see Fig. S12; the models except for the null model has similar R^2 between measured stability and predicted one to the backward model (Fig. S11).

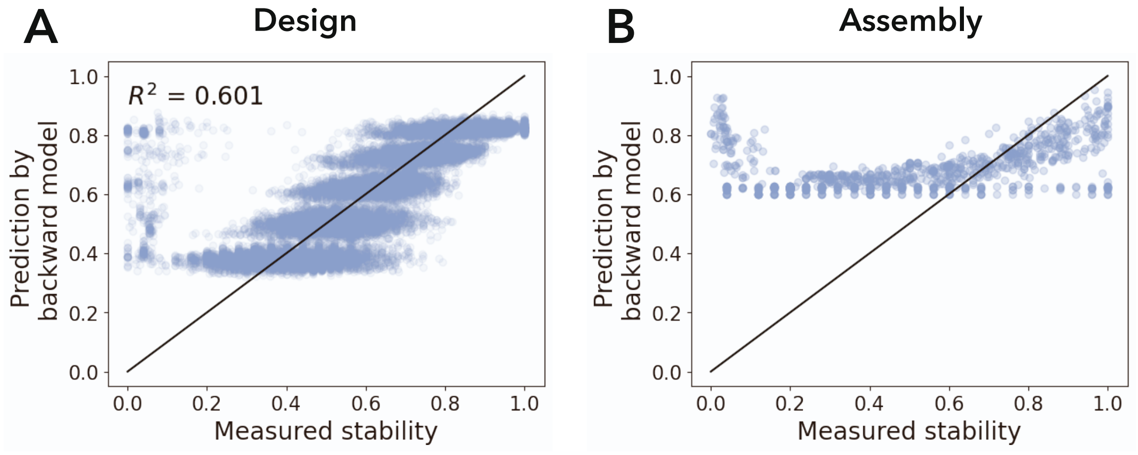

Figure S11: Predicting the stability by the backward model, related to Figure 3

The prediction of the stability of the downstream after training the backward model with the designing data. A: the competition of the true stability with the prediction by the backward model. The R-squared between the true stability and the estimated stability is 0.604. B: The same analysis but using the sub-sampled data from the assembly scenario so that we have 100 data where stability is $[0.1 \times i, 0.1 \times (i+1)]$, for each $i = 0, \ldots 9$. In both panels, the black diagonal lines represent that the model prediction perfectly matches the true stability

We emphasize that the backward model is the best predictive model, but this model does not suggest causality: we could not tell whether stability increases/decreases or not by changing some features of the communities. This is because AIC is designed to select a model with high predictability.

Table S2: Backward model, related to Figure 3

Feature	Coefficient	Standard error	P-value
Intercept	0.5189	0.016	$< 10^{-3}$
Total negative up	0.0953	0.025	$< 10^{-3}$
Total positive trans	0.0755	0.018	$< 10^{-3}$
Degree trans	-0.0944	0.026	$< 10^{-3}$
ρ	0.7134	0.017	$< 10^{-3}$
μ	-0.0587	0.016	$< 10^{-3}$

Table S3: Null model, related to Figure 3

Feature	Coefficient	Standard error	P-value
Intercept	0.4611	0.015	$< 10^{-3}$

Data S6 Details of causal inference, related to Figure 3

In the main text, we only show the effect of each controllable feature on the stability in Fig 3B. As these results come from various regression models, we explain the results of the regression models in this section. The full model of the causal diagram is given in Fig. 3A. This network suggests which feature we should include in the logistic regression analysis to satisfy the backdoor criteria[46;47;48], which tell us which features the regression model should or should not have to infer the causation from the features we are interested in. When we analyze the effect of the species richness in the upstream communities, the logistic regression model should exclude the

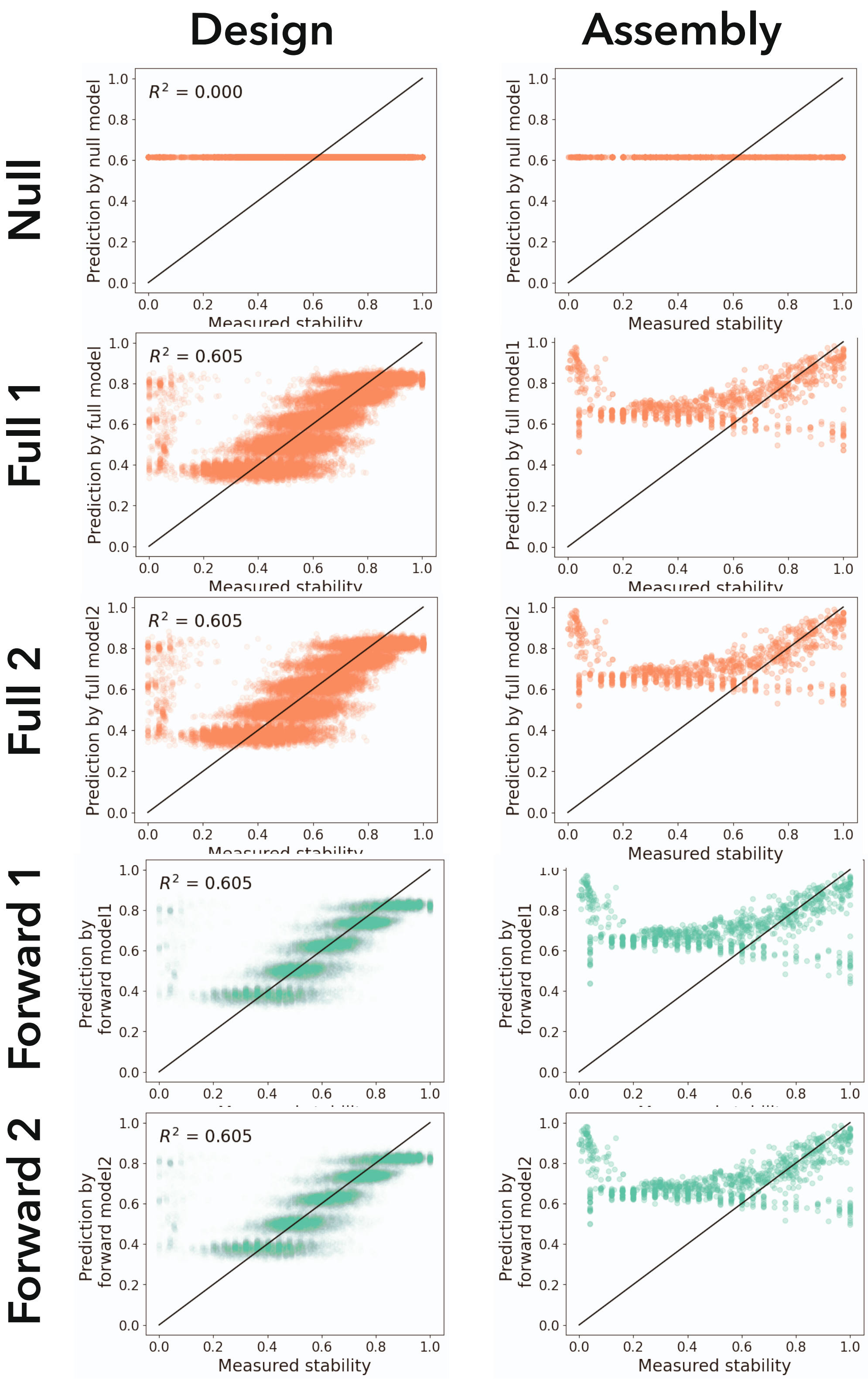

Figure S12: Prediction by the other logistic regression models, related to Figure 3

Predictions of the stability by each model are summarized. Each row corresponds to each model. The left column shows the comparisons between the true stability in the designing scenario and the prediction by each model. R^2s between the true stability and the predicted one are shown on the top left of each panel. The right panel shows the same analysis for the sub-sampled data from the assembly scenario so that we have 100 data where stability is $[0.1 \times i, 0.1 \times (i+1)]$, for each $i = 0, \ldots 9$. In each panel, the black diagonal lines represent that the model prediction perfectly matches the true stability.

Table S4: Forward model 1, related to Figure 3

Feature	Coefficient	Standard error	P-value
Intercept	0.5188	0.016	$< 10^{-3}$
Total positive up	0.0180	0.040	0.729
Mean positive up	0.0132	0.016	0.7456
Total negative up	0.0493	0.039	0.203
Mean negative up	−0.0026	0.017	0.880
Degree up	0.0700	0.054	0.195
Richness up	0.0055	0.016	0.733
Total positive down	−0.0031	0.030	0.917
Total negative down	−0.0238	0.027	0.375
Richness down	0.0132	0.016	0.412
Total positive trans	0.0661	0.032	0.040
Mean positive trans	0.0027	0.018	0.880
Total negative trans	0.0378	0.028	0.183
Mean negative trans	−0.0160	0.018	0.366
Degree trans	−0.1388	0.036	$< 10^{-3}$
ρ	0.7113	0.017	$< 10^{-3}$
μ	−0.0603	0.016	$< 10^{-3}$

total strength of positive/negative interactions within the upstream community and those from the upstream to the downstream. In contrast, when we analyze the total strength of positive/negative interactions within the upstream community and those from the upstream to the downstream, the logistic regression model should include the species richness in the upstream community. Table S9 summarizes the coefficients and the p-values in each logistic regression. Note that we can also infer the effects on the stability from the species richness in the downstream community, ρ, and μ in the three logistic regression models in Table S9, where the coefficient of each feature is almost identical across the three models. We can see that the ρ has the largest effect on the stability in the analyzed features.

We also inferred the causal effects of the total strength of positive and negative interactions in the downstream patch, respectively. The logistic regression model, in this case, included the species richness in the upstream and downstream, respectively, the total strength of positive or negative interactions in the downstream, respectively, ρ, and μ (Table S10). Although the total strength of negative interaction in the downstream is not statistically significant, the coefficient is as large as the total strength of positive interactions from the upstream to the downstream communities. This result is consistent with the previous studies[34;35;36;37], where species-rich communities have high resistance to invasion due to resource competition[38] because resource competition is implemented as negative interactions in this study.

Table S5: Full model 1, related to Figure 3

Feature	Coefficient	Standard error	P-value
Intercept	0.5189	0.016	$< 10^{-3}$
Total positive up	0.0117	0.041	0.775
Mean positive up	0.0133	0.018	0.450
Total negative up	0.0486	0.039	0.214
Mean negative up	−0.0022	0.017	0.898
Degree up	0.0722	0.056	0.198
Richness up	0.0057	0.016	0.723
Total positive down	−0.0067	0.036	0.852
Mean positive down	0.0058	0.023	0.799
Total negative down	−0.0129	0.035	0.710
Mean negative down	−0.0119	0.021	0.576
Degree down	−0.0085	0.048	0.859
Richness down	0.0131	0.016	0.414
Total positive trans	0.0692	0.034	0.042
Mean positive trans	0.0024	0.018	0.895
Total negative trans	0.0400	0.030	0.179
Mean negative trans	−0.0165	0.018	0.355
Degree trans	−0.1395	0.036	$< 10^{-3}$
ρ	0.7111	0.017	$< 10^{-3}$
μ	−0.0604	0.016	$< 10^{-3}$

Table S6: Full model 2, related to Figure 3

Feature	Coefficient	Standard error	P-value
Intercept	0.5188	0.016	$< 10^{-3}$
Total positive up	0.0372	0.035	0.293
Mean positive up	0.0089	0.017	0.608
Total negative up	0.0719	0.035	0.038
Mean negative up	−0.0064	0.017	0.709
Richness up	0.0054	0.016	0.739
Total positive down	−0.0059	0.033	0.857
Mean positive down	0.0062	0.022	0.779
Total negative down	−0.0111	0.031	0.718
Meannegativedown	−0.0127	0.021	0.551
Richness down	0.0141	0.016	0.378
Total positive trans	0.0675	0.032	0.038
Mean positive trans	0.0010	0.018	0.956
Total negative trans	0.0308	0.028	0.279
Mean negative trans	−0.0171	0.018	0.336
Degree trans	−0.1120	0.029	$< 10^{-3}$
ρ	0.7113	0.017	$< 10^{-3}$
μ	−0.0600	0.016	$< 10^{-3}$

Table S7: Forward model 2, related to Figure 3

Feature	Coefficient	Standard error	P-value
Intercept	0.5187	0.016	$< 10^{-3}$
Total positive up	0.01379	0.035	0.283
Mean positive up	0.0088	0.017	0.610
Total negative up	0.0718	0.035	0.038
Mean negative up	-0.0066	0.017	0.698
Richness up	0.0052	0.016	0.749
Total positive down	0.0010	0.030	0.974
Total negative down	-0.0199	0.027	0.454
Richness down	0.0142	0.016	0.376
Total positive trans	0.0664	0.032	0.039
Mean positive trans	0.0009	0.018	0.960
Total negative trans	0.0300	0.028	0.279
Mean negative trans	-0.0170	0.018	0.326
Degree trans	-0.1116	0.029	$< 10^{-3}$
ρ	0.7115	0.017	$< 10^{-3}$
μ	-0.0600	0.016	$< 10^{-3}$

Table S8: Model selection, related to Figure 3

Model	ΔAIC
Backward	0.000
Forward 1	5.212
Forward 2	7.728
Full 1	11.2289
Full 2	11.580
Null	3283.415

Table S9: Coefficients in causal inference upstream, related to Figure 3

feature(s) of interest	Intercept	Richness up	Total positive up	Total negative up	Total positive trans	Total negative trans	Richness down	ρ	μ
richness up	0.518 ($< 10^{-3}$)	0.006 (0.73)	N.A.	N.A.	N.A.	N.A.	0.017 (0.29)	0.708 ($< 10^{-3}$)	−0.059 ($< 10^{-3}$)
Total positive/negative up	0.518 ($< 10^{-3}$)	0.006 (0.73)	0.019 (0.50)	0.032 (0.27)	N.A.	N.A.	0.013 (0.43)	0.709 ($< 10^{-3}$)	−0.058 ($< 10^{-3}$)
Total positive/negative trans	0.518 ($< 10^{-3}$)	0.005 (0.76)	N.A.	N.A.	0.049 (0.03)	0.019 (0.39)	0.013 (0.41)	0.710 ($< 10^{-3}$)	−0.059 ($< 10^{-3}$)

In each element, the values outside the parentheses indicate the coefficients in the logistic regression while the values inside the parentheses show the p-values. N.A. represents "not applicable."

Table S10: Coefficients in causal inference downstream, related to Figure 3

feature(s) of interest	Intercept	Richness up	Richness down	Total positive down	Total negative down	ρ	μ
Total_positive/negative_down	0.518 ($< 10^{-3}$)	0.006 (0.73)	0.015 (0.34)	−0.001 (0.97)	0.045 (0.07)	0.709 ($< 10^{-3}$)	−0.058 ($< 10^{-3}$)

Table S11: Coefficient of degree trans in causal inference, related to Figure 3

feature(s) of interest	Intercept	Richness up	Richness down	Degree trans	ρ	μ
Degree_trans	0.518 ($< 10^{-3}$)	0.006 (0.73)	0.016 (0.33)	−0.013 (0.41)	0.708 ($< 10^{-3}$)	−0.058 ($< 10^{-3}$)

In the main text, we do not assume the paths from the mean degrees of the species interactions to the stability in Fig.3 because no previous studies, to our best knowledge, suggest the causation from the degree to the stability. However, as the predictive models in Data S5 (Tables S4–S7) suggest significant relations between degree trans (i.e., mean degree of species interactions from upstream to downstream species) and the stability, one may be interested in whether degree trans could affect the stability or not by assuming additional edges from Degree trans to resistance to the environmental changes and/or resistance to the invasion in Fig.3. In this new causal diagram, we can infer the causal effect of degree trans on the stability by the logistic regression model that has species richness in upstream and downstream, respectively, degree trans, ρ, and μ. However, degree_trans does not have a significant effect on the stability (Table S11): this result clarifies the difference between the prediction (in Data S5) and the causal inference. Fig. S13 shows that degree trans correlates with the total strength of positive interactions from upstream to downstream communities, which increases the stability. This would be why the predictive models in Data S5 show that degree trans has the significant effect. Notably, adding edges from degree trans to resistance to the environmental changes and/or resistance to the invasion in Fig.3 does not affect the results in Table S9 because the back door criteria are still maintained.

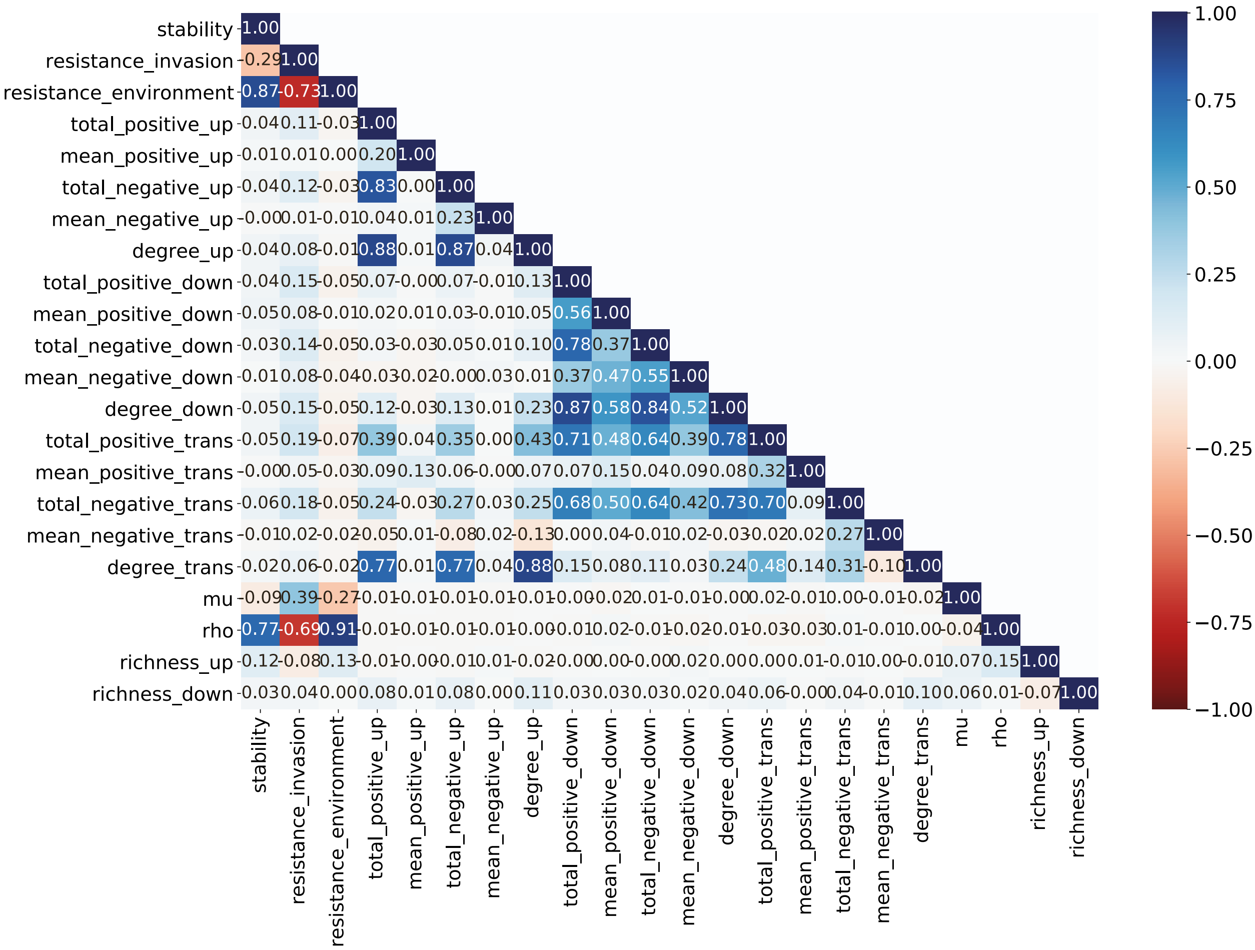

Figure S13: Pearson correlations in designing scenarios, related to Figure 3

Pearson correlations among stability, two types of resistance, and the community features. Degree_trans has strong or moderate correlations with the total strength of interactions in the upstream community or those from upstream to downstream communities, including totalpositivetrans.

Data S7 Additional manipulation of positive interactions, related to Figure 4

In the main text, we manipulated the strength of the positive effects from upstream species to downstream species to clarify whether these species interactions increase the downstream stability (Figs. 4A and B). However, to balance the mean of the off-diagonal elements of the interaction matrix, all interactions from downstream species to upstream species are assumed negative, and the strength of these negative interactions increase while the positive interactions from upstream species to downstream species get stronger. In this case, the stability would increase over the strength of positive interactions from upstream species to downstream species because upstream species receive stronger negative effects and cannot colonize when they invade the downstream patch (i.e., larger resistance to invasion), but not because of the positive effects from upstream species to downstream species.

To remove this bias, we increased the strength of the positive interactions from upstream species to downstream species while assuming species interaction coefficients of the opposite direction in two ways: (i) fixing the species interaction coefficients zeros, or (ii) sampling the coefficients from the normal distribution whose mean is 0 and the standard deviation is 0.25. Remarkably, these two manipulations break the assumption that interspecific interactions follow the normal distribution whose mean is zero, see Eq (S7).

In the first case, upstream species can colonize the downstream patch when their basal growth rates in the downstream patch are larger than in the upstream patch ($r_{i2} > r_{i1}$). This is because such invaders would not receive negative interactions within the downstream communities. In this case, some simulations shown negative or no changes in the stability over the strength of positive interactions from upstream species to downstream species (Fig. S14A). This result indicates that the increase of negative interactions from downstream species to upstream species increases the stability in Fig. 4. However, about half simulations (52 out of 110 simulations) show positive correlations between the strength of the positive interactions and the stability (Fig. S14B). In addition, one-sided Wilcoxon signed-rank test suggests that the correlation coefficients are biased toward positive ($W = 1620$, $p = 0.042$; rank-biserial correlation is 0.23). These results support our conclusion that the positive interactions from upstream species to downstream species stabilize the downstream community.

In the second manipulation, we observed similar patterns of changes in stability over the strength of positive interactions from upstream to downstream (Fig. S14C): we can observe negative or no changes in the stability. Although one-sided Wilcoxon signed rank test does not suggest statistical significance ($W = 2488.5$, $p = 0.166$; rank-biserial correlation is 0.11) in this case, Fig. S14D shows more positive correlations (50 of 110 meta-communities) than negative ones (44 of 110).

The difference among Figs. 4 and S14 highlights the effect of species interactions from downstream species to upstream one. Because these interactions cannot realize unless upstream species invade downstream, our analyses in the main text do not include these interactions. However, the effect of interactions from downstream species to upstream species would be straightforward: if downstream species have more positive (negative) interactions to upstream species, the upstream species are (un)likely to establish once they invade the downstream

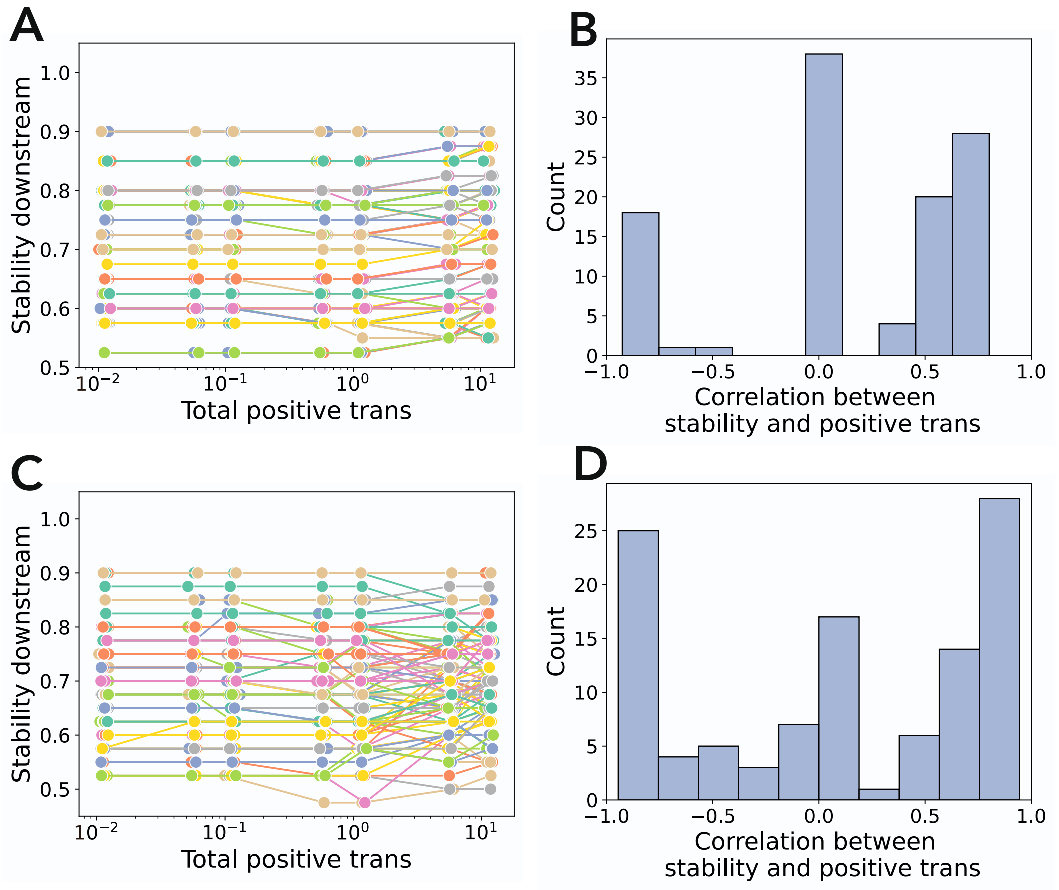

Figure S14: Manipulation of positive effects while fixing negative effects, related to Figure 4

We manipulate the strength of positive interactions from upstream species to downstream species while (A and B) fixing the strength of the interactions in the opposite direction zero or (C adn D) sampling the interaction coefficients from $\mathcal{N}\left(0, 0.25^2\right)$. A and C: We plotted the downstream stability over the strength of positive interactions from upstream species to downstream species. Each line corresponds to one of 110 meta-communities. B and D: The histogram of correlation coefficients between the strength of the positive interactions and the stability in panels A and C, respectively are shown. In panel B, 52 meta-communities show positive correlations, 20 show negative correlations, and 38 show no correlations, while 50 meta-communities show positive correlations, 44 show negative correlations, and 16 show no correlationsin panel D .

patch, which indicates low (high) downstream stability. Indeed, in the main text of Figs. 4A and B, the downstream species have only negative interactions to the upstream species. In contrast, such interactions are neutral in Figs. S14A and B, and can be positive in Figs. S14C and D. Therefore, we suggest that these unrealized interactions (i.e., from downstream species to upstream species) can also affect the downstream stability.

Data S8 Adding or removing upstream species, related to Figure 4

In this section, we sought to manipulate the total strength of positive interactions from up- to downstream communities by adding or removing one species in the assembly scenario ($n = 20308$, and 4023, respectively). Changing species richness would be an intuitive way of changing total interaction strength: adding species increases the total strength of the positive interactions while removing species decreases it.

We added/removed one species to/from the upstream communities which experienced 10 migration events in

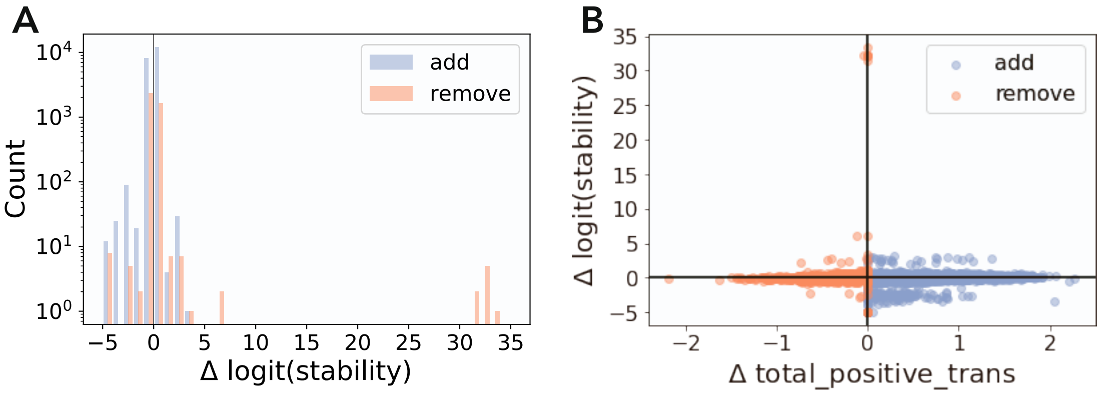

Figure S15: Manipulation of upstream species richness, related to Figure 4

We manipulated the total strength of positive interactions from the upstream to the downstream communities by adding/removing one species to/from the upstream communities in the assembly scenario. A: Introducing species tends to increases the stability ($n = 20308$, median of changes in the logit function of the stability: 0.018, one-sided Wilcoxon signed-rank test: $U = 117072109.5$ and $p = 4.8 \times 10^{-88}$) while removing species decrease the stability ($n = 4023$, median of changes in the logit function of the stability: -0.031, one-sided Wilcoxon signed-rank test: $U = 3199368$ and $p = 7.9 \times 10^{-31}$)). B: By adding or removing species, we can also manipulate the total strength of positive interactions from the upstream and the downstream communities, which positively correlate with the changes in the stability (Spearman correlation: $\rho = 0.15$, and $p < 3.0 \times 10^{-127}$). However, this correlation is very weak, and increasing/decreasing the positive interactions does not necessarily increase/decrease the stability, respectively.

the assemble scenario because the variation of the stability at the time step is large and the stability is unlikely to be one (see Fig. S2). Then, we calculated how adding or removing species changed the stability and the total strength of positive interactions from the upstream to the downstream communities. We also calculated Spearman correlation coefficients between the stability and the total strength of positive interactions.

Fig. S15A shows that introducing species tends to slightly increase downstream stability (median change in the logit of stability: 0.018, one-sided Wilcoxon signed-rank test: $W = 117072109.5$ and $p = 4.8 \times 10^{-88}$; rank-biserial correlation is 0.16), while removing species slightly decreases it (median change in the logit of stability: -0.031, one-sided Wilcoxon signed-rank test: $T = 3199368$ and $p = 7.9 \times 10^{-31}$; rank-biserial correlation is -0.21). In this data set, the change in the total strength of positive interactions from up- to downstream positively correlated with the change in stability but the correlation was weak (Spearman correlation coefficient: 0.15, $p < 10^{-126}$, Fig. S15B). This may be because adding or removing species changes community features other than the feature of interest (e.g., the total strength of positive and negative interactions within the upstream communities).
